# Supplementary material for: Particle exchange statistics beyond fermions and bosons
Source: Nature. 2025 Jan 8;637(8045):314–8. doi: 10.1038/s41586-024-08262-7 (PMC11711093; doi:10.1038/s41586-024-08262-7)
Supplement: Supplementary file 1 — Supplementary Sections 1–6, which include Supplementary Text and Data, Supplementary Table 1 and Supplementary Figs. 1–7. [file 41586_2024_8262_MOESM1_ESM.pdf]

nature portfolio

https://doi.org/10.1038/s41586-024-08262-7

Supplementary information

Particle exchange statistics beyond fermions and bosons

In the format provided by the authors and unedited

Supplementary Information for "Particle exchange statistics beyond fermions and bosons"

Zhiyuan Wang[1,2,3] and Kaden R. A. Hazzard[1,2]
[1]Department of Physics and Astronomy, Rice University, Houston, Texas 77005, USA
[2]Rice Center for Quantum Materials, Rice University, Houston, Texas 77005, USA
[3]Max-Planck-Institut für Quantenoptik, Hans-Kopfermann-Str. 1, 85748 Garching, Germany
(Dated: December 4, 2024)

This Supplementary Information fills in several technical details omitted in the main text. In Sec. S1 we explain the difference between parastatistics and other known types of particle statistics, and the difference between our theory of parastatistics and Green's theory [1]. In Sec. S2 we present the detailed construction of the state space and derive the generalized exclusion statistics for all the R-matrices that appeared in the main text, and prove that our second quantization theory is well-defined for an arbitrary R-matrix, including non-unitary ones. In Sec. S3 we show the relation between the second quantization formulation of parastatistics and the wavefunction formulation we discussed in the introduction part of the main text. In Sec. S4 and Sec. S5 we present the detailed construction of the 1D solvable spin model defined in Eq. (17) and the 2D solvable spin model defined in Eq. (32), respectively, including the definition of local operators $\{\hat{x}^{\pm}_{i,a}, \hat{y}^{\pm}_{i,a}\}_{a=1}^{m}$ and the tensors u_L, v_L, u_R, v_R, and we prove that the MPO JWT defined in Eq. (18) and Eq. (34) map the spin Hamiltonians into free paraparticle Hamiltonians. Finally in Sec. S6 we discuss superselection rules and the observability of elementary paraparticles.

S1. DIFFERENCE BETWEEN PARASTATISTICS AND OTHER TYPES OF PARTICLE STATISTICS

In the following, we briefly explain the difference between parastatistics and other known types of particle statistics, including non-Abelian anyons [2], parafermions [3], and other types of exclusion statistics. We also explain the difference between our theory of parastatistics and Green's theory [1].

For non-Abelian anyons in 2D, braiding two anyons also results in a matrix rotation on the internal space (more precisely, the topologically protected degenerate fusion space) of a system with multiple anyonic excitations, similar to Eq. (3). However, for non-Abelian anyons, the matrices $\{R_j\}_{j=1}^{n-1}$ only satisfy the second equation in Eq. (4), but not the first one, and this is the reason why anyons cannot be consistently defined in 3+1D, where exchanging two identical particles twice should give back the original state. In 2D, parastatistics can be considered as a special case of non-Abelian statistics satisfying $R_j^2 = \mathbb{1}$; however, to the best of our knowledge, this special case was previously believed to contain only fermions and bosons. Another key difference between anyons and paraparticles is that there does not exist an exactly solvable free particle theory for genuine anyons (meaning that $R_j^2 \neq \mathbb{1}$). By a "solvable free particle theory of anyons", we mean a many-body Hamiltonian describing a system of non-interacting anyons freely moving in space, similar to Eq. (15), such that one can obtain the exact many body spectrum by solving the one particle spectrum.

Parafermions [3, 4] are exotic emergent excitations that appear in 1D $\mathbb{Z}_n$-symmetric quantum spin chains and the 1D boundary of certain 2D topological phases, such as quantum Hall/superconductor hybrids [4]. They are defined by the following algebraic relations between parafermion operators that generalize the Clifford algebra of Majorana fermions:

$$\begin{aligned} \hat{\psi}_i\hat{\psi}_j &= \omega\hat{\psi}_j\hat{\psi}_i, \text{ for } i<j, \\ \hat{\psi}_i^n &= 1, \ \forall i, \end{aligned} \tag{S1}$$

where $n \geq 2$ is an integer and ω is a primitive n-th root of unity. As we can see, despite the similarity in the names, parafermions are defined by a very different generalization of the second quantization of fermions compared to our generalization in Eq. (6), and the requirement $i<j$ in Eq. (S1) can only be consistently specified in 1D, so parafermions are intrinsically limited to 1D. Interestingly, for parafermions in 1D, there also exists an exactly solvable free particle theory [3], where one can exactly obtain the full many-body spectrum by solving the single particle spectrum. However, the solvable Hamiltonians for free parafermions are always non-Hermitian and have complex energy spectrum.

Green's theory of parastatistics [1, 5–9] is defined by a set of trilinear CRs between paraparticle creation and annihilation operators

$$\begin{aligned} \left[\left[\hat{\psi}_k^\dagger, \hat{\psi}_l\right]_\pm, \hat{\psi}_m\right]_- &= -2\delta_{km}\hat{\psi}_l, \\ \left[\left[\hat{\psi}_k, \hat{\psi}_l\right]_\pm, \hat{\psi}_m\right]_- &= 0, \end{aligned} \tag{S2}$$

where $[\hat{A}, \hat{B}]_\pm = \hat{A}\hat{B} \pm \hat{B}\hat{A}$. This theory of parastatistics is also consistently defined in any spatial dimension, and it was shown in Ref. [9] that the exchange statistics of paraparticles in this theory also realize higher dimensional representations of the symmetric group S_N, in the sense of Eqs. (3,4). Green's theory also includes

exactly solvable theories of free paraparticles, but the exclusion statistics of Green's paraparticles is much harder to compute and is still not fully solved to date [10]. In particular, unlike in our formulation, in Green's theory, the grand partition function of the whole system does not factorize as a product of single mode partition functions [10], making it challenging to compute its thermodynamic properties. Furthermore, it is not known whether the paraparticles defined by Green's theory can appear as emergent quasiparticles in condensed matter systems, in a way distinct from fermions and bosons.

In Tab. S1 we summarize the comparison between the above three types of particle statistics and the R-matrix parastatistics we introduced in the main text. A caveat about terminology is that one should be careful about the use of "paraboson" and "parafermion" in the recent literature – some of them [11–16] refer to Green's parastatistics [1] defined by Eq. (S2), some refer to parafermions [3, 4] defined by Eq. (S1), and some refer to the q-deformed bosons and fermions [17]. The last one is limited to 1D, defined by q-deformed second quantization algebra of bosons and fermions, and does not contain free particle theories.

Furthermore, some generalizations of the Pauli exclusion principle have been proposed in the first quantization formulation. Ref. [18] studied the thermodynamics of an ideal gas composed of particles with exclusion statistics $d_l = 1$ for $0 \le l \le m$, and $d_l = 0$ for $l > m$. Ref. [19] proposed a definition of exclusion statistics that is well-defined in any spatial dimension and generalizes Pauli's principle, which applies to many interesting physical systems such as quasiparticles of the fractional quantum Hall effect [20], spinons in gapless spin-1/2 antiferromagnetic chains [19], and quasiparticles in conformal field theory spectra [21, 22]. The generalization of Pauli's principle in Ref. [19] is not compatible with second quantization beyond the simple case of fermions and bosons [19]. In particular, the state counting formula in Eq. (3) of Ref. [19] does not apply to paraparticles introduced in this paper.

S2. THE STATE SPACE AND EXCLUSION STATISTICS FOR A GENERAL R-MATRIX

In the main text we derived the generalized exclusion statistics for the R-matrix in Ex. 3 in Tab. I, and constructed an orthonormal basis for the many particle state space in Eq. (11). In this section we generalize these results to an arbitrary R-matrix. Specifically, in Sec. S2 A we construct a basis for the state space for an arbitrary R-matrix, which is orthonormal if R is unitary, in Sec. S2 B we define the action of the paraparticle creation and annihilation operator $\hat{\psi}^{\pm}_{i,a}$ on these basis states, and in Sec. S2 C we calculate exclusion statistics for other R-matrices we studied in the main text. In Sec. S2 D we construct the state space in a rigorous mathematical framework, and in particular, in Sec. S2 D 5 we show that even with a non-unitary R-matrix, we can still define a Hermitian inner product on the state space, with respect to which all physical observables are Hermitian.

A. A basis for the state space

Before diving into details, let us first summarize the basic idea behind the construction of the state space in a nutshell (this idea is formalized in a rigorous mathematical framework in Sec. S2 D). Analogous to the Fock space of fermions and bosons, there is a vacuum state $|0\rangle$ satisfying $\hat{\psi}^{-}_{i,a}|0\rangle = 0$, and we always assume it is normalized as $\langle 0|0\rangle = 1$. The many-particle state space is spanned by all states of the form $|\psi\rangle = \hat{\psi}^{+}_{i_1,a_1}\hat{\psi}^{+}_{i_2,a_2}\dots\hat{\psi}^{+}_{i_n,a_n}|0\rangle$. (Note that these are not all linearly independent.) The second relation in Eq. (6) imposes linear dependence relations on these states, which gives rise to the generalized exclusion statistics. The action of creation operators on this set of states is described straightforwardly by $\hat{\psi}^{+}_{i,a}|\psi\rangle = \hat{\psi}^{+}_{i,a}\hat{\psi}^{+}_{i_1,a_1}\dots\hat{\psi}^{+}_{i_n,a_n}|0\rangle$. The action of annihilation operators $\hat{\psi}^{-}_{i,a}$ is uniquely determined by the first relation in Eq. (6), which allows us to move $\hat{\psi}^{-}_{i,a}$ all the way to the right until it hits $|0\rangle$ (which it annihilates).

However, to actually compute the exclusion statistics and to work out an explicit matrix representation of $\hat{\psi}^{\pm}_{i,a}$, we need to first construct a basis for the state space, in a way that generalizes Eq. (11) in the main text to an arbitrary R-matrix. This is what we do in the following.

Let $\{\Psi^{\alpha}_{a_1 a_2 \dots a_n}\}_{\alpha=1}^{d_n}$ be a complete set of linearly independent solutions to the system of linear equations

$$\sum_{a'_j, a'_{j+1}} R^{a_j a_{j+1}}_{a'_j a'_{j+1}} \Psi_{a_1 \dots a'_j a'_{j+1} \dots a_n} = \Psi_{a_1 \dots a_j a_{j+1} \dots a_n} \quad \text{(S3)}$$

for $j = 1, 2, \dots, n-1$. Intuitively, Eq. (S3) requires that $\Psi_{a_1 \dots a_n}$ is an R-symmetric function, which in the case of fermions or bosons ($R = \pm 1$) reduces to totally symmetric or antisymmetric functions. A basis for the state space is constructed as the set of states of the form

$$|{}^{\alpha_1}_{n_1}, {}^{\alpha_2}_{n_2}, \dots, {}^{\alpha_N}_{n_N}\rangle = \hat{\Psi}^{(1)+}_{n_1,\alpha_1} \hat{\Psi}^{(2)+}_{n_2,\alpha_2} \dots \hat{\Psi}^{(N)+}_{n_N,\alpha_N} |0\rangle, \quad \text{(S4)}$$

where the numbers $\{(n_i, \alpha_i)\}_{i=1}^{N}$ can be chosen independently for different modes (with the only constraint being $1 \le \alpha_i \le d_{n_i}$ for each i), and the operator

$$\hat{\Psi}^{(i)+}_{n,\alpha} \equiv \frac{1}{\sqrt{n!}} \sum_{a_1 a_2 \dots a_n} \Psi^{\alpha}_{a_1 a_2 \dots a_n} \hat{\psi}^{+}_{i,a_1} \hat{\psi}^{+}_{i,a_2} \dots \hat{\psi}^{+}_{i,a_n}, \quad \text{(S5)}$$

creates a multiparticle state in the mode i with occupation number n (eigenvalue of $\hat{n}_i \equiv \hat{e}_{ii}$).

When the R-matrix is unitary, we can consistently set $\hat{\psi}^{+}_{i,a} = (\hat{\psi}^{-}_{i,a})^{\dagger}$ [24] and in this case, we require that the coefficients $\{\Psi^{\alpha}_{a_1 a_2 \dots a_n}\}_{\alpha=1}^{d_n}$ are normalized as

$$\sum_{a_1, a_2, \dots, a_n} \Psi^{\beta *}_{a_1 a_2 \dots a_n} \Psi^{\alpha}_{a_1 a_2 \dots a_n} = \delta_{\alpha\beta}. \quad \text{(S6)}$$

Particle statistics	Non-Abelian anyons [2]	Parafermions [3, 4]	Green's parastatistics [1]	R-matrix parastatistics
Definition	Braided fusion category	Eq. (S1)	Eq. (S2)	Eq. (6)
Braid group	$R_j^2 \neq \mathbb{1},\ B_n$	-	$R_j^2 = \mathbb{1},\ S_n$	$R_j^2 = \mathbb{1},\ S_n$
Spatial dimension	$d = 2$	$d = 1$	Any d	Any d
Free particle theory	No	Non-Hermitian	Hard to compute thermodynamics	Yes
Emergent	Yes	Yes	Unknown	Yes

TABLE S1. Comparison between different types of particle statistics. The R-matrix parastatistics refers to the one we introduced in the main text. The last row indicates whether a given type of particle statistics is known to emerge in locally interacting spin models.

Then the basis in Eq. (S4) is orthonormal, i.e.

$$\left\langle {}^{\beta_1}_{n_1'}, {}^{\beta_2}_{n_2'}, \ldots, {}^{\beta_N}_{n_N'} \middle| {}^{\alpha_1}_{n_1}, {}^{\alpha_2}_{n_2}, \ldots, {}^{\alpha_N}_{n_N} \right\rangle = \prod_{j=1}^{N} \delta_{n_j n_j'} \delta_{\alpha_j \beta_j}, \quad \text{(S7)}$$

see Sec. S2 B for a proof.

For non-unitary R-matrices, we show in Sec. S2 D that the basis states in Eq. (S4) are still linearly independent, and we define an alternative Hermitian inner product on the state space [which is generally different from Eq. (S7)], with respect to which all physical observables are still Hermitian.

B. Action of $\hat{\psi}^{\pm}_{i,a}$ on the basis states

The actions of $\{\hat{\psi}^{\pm}_{i,b} | 1 \leq b \leq m, 1 \leq i \leq N\}$ on the basis states in Eq. (S4) are uniquely determined by the fundamental CRs in Eq. (6): the action of an annihilation operator $\hat{\psi}^{-}_{i,a}$ is determined by using the first relation in Eq. (6) to move $\hat{\psi}^{-}_{i,a}$ all the way to the right until it hits $|0\rangle$, and the action of a creation operator $\hat{\psi}^{+}_{i,a}$ is determined by using the second relation in Eq. (6) to move it to the right, passing all the $\hat{\psi}^{+}_{j,b}$ for $j < i$, and then combine it with $\hat{\Psi}^{+}_{n_i,\alpha}$.

It turns out that the resulting matrix representation of $\{\hat{\psi}^{\pm}_{i,b}\}$ obtained this way is equivalent to using the MPO JWT in Eq. (18) to represent $\{\hat{\psi}^{\pm}_{i,b}\}$ as MPO string operators acting on a 1D spin chain, where the local operators $\hat{y}^{\pm}_{ja}$ and $\hat{T}^{\pm}_{j,ab}$ are explicitly defined in Sec. S4 A. This is reminiscent of the familiar fact in the second quantization of fermions that the action of fermionic operators on the particle number basis involves a fermion minus sign, and implementing such a fermion minus sign computationally is equivalent to doing a JWT. For paraparticles, the fermion minus sign becomes the paraparticle R-matrix, and the JW string becomes an MPO string of R [or more precisely the tensor $T^{\pm}$, which is constructed out of R and $\{\Psi^{\alpha}_{a_1 a_2 \ldots a_n}\}_{\alpha=1}^{d_n}$ in Eq. (S28)].

The MPO representation of $\{\hat{\psi}^{\pm}_{i,b}\}$ allows us to prove the orthonormality condition in Eq. (S7): under the MPO JWT, the basis state $|{}^{\alpha_1}_{n_1}, {}^{\alpha_2}_{n_2}, \ldots, {}^{\alpha_N}_{n_N}\rangle$ of the Fock space defined in Eq. (S4) is mapped to the product state $|n_1, \alpha_1\rangle \otimes |n_2, \alpha_2\rangle \otimes \ldots \otimes |n_N, \alpha_N\rangle$ of the 1D spin chain, and the latter is orthonormal since the local basis $|n, \alpha\rangle$ of the on-site Hilbert space is shown to be orthonormal in Sec. S4 A.

C. Calculation of exclusion statistics and single mode partition functions

1. R-matrices in Tab. S1

We here present the calculation of the numbers $\{d_n\}_{n\geq 0}$ for the R-matrices in Exs. 1-4. To this end, we need to solve Eq. (S3) for each R-matrix and for each particle number n. Note that Eq. (S3) does not put any restriction on Ψ for $n = 0$ and $n = 1$, so we have $d_0 = 1, d_1 = m$ for all the four families of R-matrices. The physical meaning of this is clear: we always have one vacuum state $|0\rangle$, and m degenerate single particle states. For the R-matrix in Ex. 1, Eq. (S3) sets the requirement that $\Psi_{a_1 a_2 \ldots a_n}$ is antisymmetric under the exchange of any two neighboring indices, e.g., $\Psi_{a_1 a_2 \ldots a_n} = -\Psi_{a_2 a_1 \ldots a_n}$. Therefore for each n, Ψ has $\binom{m}{n}$ independent components, which can be chosen to be $\{\Psi_{a_1 a_2 \ldots a_n} \mid 1 \leq a_1 < a_2 < \ldots < a_n \leq m\}$, therefore, $d_n = \binom{m}{n}$ for $0 \leq n \leq m$ and $d_n = 0$ for $n > m$. For the R-matrix in Ex. 2, Eq. (S3) still relates an arbitrary component $\Psi_{a_1 a_2 \ldots a_n}$ to an element in $\{\Psi_{a_1 a_2 \ldots a_n} \mid 1 \leq a_1 < a_2 < \ldots < a_n \leq m\}$, although potentially with a different sign factor, and we still have $\Psi_{a_1 a_2 \ldots a_n} = 0$ if any two indices are equal. This leads to the same d_n as in Ex. 1. For the R-matrix in Ex. 3, Eq. (S3) becomes $\Psi_{a_1 a_2 \ldots a_n} = -\Psi_{a_1 a_2 \ldots a_n}$, leading to $\Psi = 0$ and therefore $d_n = 0$ for any $n \geq 2$. For the R-matrix in Ex. 4, Eq. (S3) with $n = 2$ gives $\lambda_{ab} \sum_{c,d} \xi_{cd} \Psi_{cd} = 2\Psi_{ab}$, and since $\mathrm{Tr}[\lambda \xi^T] = 2$, this equation has a unique solution $\Psi_{ab} = \lambda_{ab}$ (up to a constant factor), therefore $d_2 = 1$. Moreover, Eq. (S3) with $n = 3$ implies $\Psi_{abc} = \lambda_{ab}\phi_c = \phi'_a \lambda_{bc}$ for some vectors ϕ, ϕ', which has no nonzero solution since λ is invertible, leading to $d_n = 0$ for $n \geq 3$ (the case for $n > 3$ is proved by applying this argument to the first 3 indices of $\Psi_{a_1 a_2 \ldots a_n}$).

The single mode partition function $z_R(x)$ can be calculated directly from the definition in Eq. (12), the results are given in Tab. I. In the mathematics literature $z_R(x)$ (where $x = e^{-\beta\epsilon}$) is called the *Hilbert series* of the R-matrix [25]. There is a very useful identity relat-

ing the Hilbert series of the R-matrices R and $-R$ (note that $-R$ also satisfies the YBE in Eq. (5) if R does): $z_R(-x)z_{-R}(x) = 1$, which allows us to compute the exclusion statistics $\{d_n\}_{n\geq 0}$ of $-R$ if the exclusion statistics of R is known. For example, for the R-matrix in Ex. 4, we have $z_{-R}(x) = 1/(1-mx+x^2)$, from which we obtain $d_0 = 1, d_1 = m, d_2 = m^2 - 1$, and $d_{n+1} = md_n - d_{n-1}$ for $n \geq 1$.

2. *The set-theoretical R-matrix in Eq.* (29)

The single mode partition function $z_R(x) = (1+x)^4$ of the set-theoretical R-matrix defined in Eqs. (29,31) can be proved either by directly solving Eq. (S3), or by using the following fact

Fact S2.1. (Proposition 1.7 in Ref. [26]) Consider R as a quantum gate acting on two neighboring qudits ($d = m = 4$), and consider an arbitrary quantum circuit generated by the gates $R_{12}, R_{23}, \ldots, R_{n-1,n}$ acting on a system of n qudits. Then there exists a unitary transformation $\hat{U}$ that simultaneously transforms $R_{12}, R_{23}, \ldots, R_{n-1,n}$ into the trivial swap gates, i.e.

$$UR_{j,j+1}U^\dagger = -X_{j,j+1},\ j = 1, 2, \ldots, n-1, \tag{S8}$$

where X is the two qudit swap gate. Such a unitary transformation can be constructed from Eq. (1.16) in Ref. [26]

$$U|x_1, \ldots, x_n\rangle = |f_{x_n}f_{x_{n-1}\cdots f_{x_2}}(x_1), \ldots, f_{x_n}(x_{n-1}), x_n\rangle, \tag{S9}$$

where $x_1, x_2, \ldots, x_n \in \{1, 2, 3, 4\}$ label basis states of the n qudits, and $f_y(x)$ is the second component of $r(x, y)$. Therefore we have $z_R(x) = z_{-X}(x) = (1+x)^4$.

D. Mathematical details on the structure of the state space

The goal of this section is to provide a rigorous mathematical framework for our second quantization formulation of parastatistics, and in the process we prove some technical claims we made in the previous sections and in the main text. It can be skipped by most readers without impacting the understanding of physics.

In this section we construct the state space and define the action of $\hat{\psi}^\pm_{i,a}$ within an alternative, rigorous mathematical framework that analyzes the structure of the second quantization algebra [defined by Eq. (6) of the main text] and its representations. This framework proves several technical claims we made in the previous sections and the main text. Specifically, Secs. S2 D 2-S2 D 4 establishes that (1) the action of $\hat{\psi}^\pm_{i,b}$ described in Sec. S2 B is an irreducible representation of the second quantization algebra (6), and it is the unique irreducible representation subject to some physical constraints; (2) the basis states constructed in Eq. (S4) are linearly independent even for non-unitary R-matrices. Furthermore, in Sec. S2 D 5 we show that even with a non-unitary R-matrix, we can still define a Hermitian inner product on the state space, with respect to which all physical observables are Hermitian.

1. *Notations and definitions*

We begin by introducing some notations. Denote by $\mathcal{X}_{R,N}$ the unital associative algebra over $\mathbb{C}$ generated by $\{\hat{\psi}^\pm_{i,b}|1 \leq i \leq N, 1 \leq b \leq m\}$ modulo all the relations in Eq. (6). Define $\mathcal{X}^+_{R,N}$ as the (unital) subalgebra of $\mathcal{X}_{R,N}$ generated by all the creation operators $\{\hat{\psi}^+_{i,b}|1 \leq i \leq N, 1 \leq b \leq m\}$, and similarly $\mathcal{X}^-_{R,N}$ the (unital) subalgebra of $\mathcal{X}_{R,N}$ generated by all the annihilation operators $\{\hat{\psi}^-_{i,b}|1 \leq i \leq N, 1 \leq b \leq m\}$.

An important observation is that the algebra $\mathcal{X}_{R,N}$ can be obtained from $\mathcal{X}_{R,1}$ as

$$\mathcal{X}_{R,N} \cong \mathcal{X}_{\Pi\boxtimes R,1}, \tag{S10}$$

where $\Pi \boxtimes R$ is the direct product R-matrix defined as

$$(\Pi \boxtimes R)^{AB}_{CD} \equiv \Pi^{ij}_{kl} R^{ab}_{cd}, \tag{S11}$$

where we group the spatial index $i = 1, 2, \ldots, N$ and the internal index a in $\hat{\psi}^\pm_{i,a}$ into a single collective index: $A = (i, a), B = (j, b), C = (k, c)$ and $D = (l, d)$. Π acts on the spatial part defined as $\Pi^{ij}_{kl} = \delta_{il}\delta_{jk}$, and R acts on the internal part. It is straightforward to check that $\Pi\boxtimes R$ constructed this way also satisfies the YBE Eq. (5), and Eq. (S10) can be checked by comparing the defining CRs of both sides. For this reason, in Secs. S2 D 2 and S2 D 3 we focus on the algebra $\mathcal{X}_R \equiv \mathcal{X}_{R,1}$, but keep in mind that any claim we make on $\mathcal{X}_R$ applies equally well to $\mathcal{X}_{R,N}$ by using the product R-matrix $\Pi \boxtimes R$. We will omit the mode labels i, j and simply write $\hat{\psi}^\pm_a$ when there is no confusion.

2. *Existence and uniqueness of vacuum state from physical requirements*

For the theory to make physical sense, the spectrum of the total particle number operator $\hat{n}$ should be bounded from below. This means that there exists at least one state $|n_{\min}\rangle$ with the smallest eigenvalue $n_{\min}$ of $\hat{n}$. Since $\hat{\psi}^-_a$ decreases the eigenvalue of $\hat{n}$ by 1, the minimality of $n_{\min}$ requires that $\hat{\psi}^-_a|n_{\min}\rangle = 0, \forall a$, since otherwise $\hat{\psi}^-_a|n_{\min}\rangle$ would be an eigenstate of $\hat{n}$ with eigenvalue $n_{\min} - 1 < n_{\min}$. Therefore $\hat{n}|n_{\min}\rangle = \sum_a \hat{\psi}^+_a\hat{\psi}^-_a|n_{\min}\rangle = 0$, i.e., $n_{\min} = 0$. We call this state the vacuum state, denoted by $|0\rangle$.

It can be proven that in an irrep V of $\mathcal{X}_R$, the vacuum state must be unique. Here is a sketch of the proof by contradiction: assume there exists two linearly independent vacuum states, say $|0\rangle, |0'\rangle \in V$. Then $V_0 = \mathcal{X}^+_R|0\rangle$

would be invariant under the action of $\mathcal{X}_R$. To prove this, it is enough to show that V_0 is invariant under all the generators $\hat{\psi}_a^{\pm}$ of $\mathcal{X}_R$: $\hat{\psi}_a^+$ leaves V_0 invariant since $\hat{\psi}_a^+\mathcal{X}_R^+ \subseteq \mathcal{X}_R^+$, while $\hat{\psi}_a^-\mathcal{X}_R^+ \subseteq \mathcal{X}_R^+\hat{\psi}_a^- + \mathcal{X}_R^+$ according to the first relation in Eq. (6), so $\hat{\psi}_a^- V_0 \subseteq V_0$. Therefore, V_0 is a subrepresentation of V. Furthermore, $|0'\rangle \notin V_0$ since the only state in V_0 annihilated by $\hat{n}$ is $|0\rangle$. Therefore, V_0 is a proper subrepresentation of V, contradicting the irreducibility of V.

3. *The state space generated by $|0\rangle$ and $\{\hat{\psi}_a^+\}$*

The algebra $\mathcal{X}_R$ is the special case of the quantum Weyl algebras (QWAs) $A_m(R)$ studied in Ref. [27] with $q = 1$, by identifying x_a with $\hat{\psi}_a^+$ and ∂_a with $\hat{\psi}_a^-$, and Thm. 1.5 in Ref. [27] provides the rigorous mathematical foundation for the construction of state space:

Theorem S2.1. (Thm. 1.5 in Ref. [27]) There is a vector space isomorphism $\mathbb{C}_R\langle\hat{\psi}_a^+\rangle \otimes \mathbb{C}_R\langle\hat{\psi}_a^-\rangle \cong \mathcal{X}_R$, where $\mathbb{C}_R\langle\hat{\psi}_a^+\rangle$ is the unital associative algebra generated by $\{\hat{\psi}_b^+ | 1 \le b \le m\}$, subject to the second relations in Eq. (6), and similarly $\mathbb{C}_R\langle\hat{\psi}_a^-\rangle$ is the unital associative algebra generated by $\{\hat{\psi}_b^- | 1 \le b \le m\}$, subject to the third relations in Eq. (6).

This theorem extends the simpler fact that, as a vector space, $\mathcal{X}_R$ is spanned by $\mathcal{X}_R^+ \otimes \mathcal{X}_R^-$, since for any monomial of $\hat{\psi}_1^+, \ldots, \hat{\psi}_m^+, \hat{\psi}_1^-, \ldots, \hat{\psi}_m^-$ in $\mathcal{X}_R$ (e.g. $\hat{\psi}_a^-\hat{\psi}_b^+\hat{\psi}_c^-\hat{\psi}_d^+$), one can always use the first relation in Eq. (6) to "normal order" all $\hat{\psi}_a^+$s to the left and $\hat{\psi}_a^-$s to the right, leading to a sum of terms, each with at most m creation and m annihilation operators. The non-trivial aspect of this theorem is that $\mathcal{X}_R^+ \cong \mathbb{C}_R\langle\hat{\psi}_a^+\rangle$, and $\mathcal{X}_R^- \cong \mathbb{C}_R\langle\hat{\psi}_a^-\rangle$, i.e. the relations in the first and third lines of Eq. (6) do not imply any additional relations on the $\hat{\psi}_a^+$s other than the second line in Eq. (6). See Ref. [27] for a detailed proof.

We now construct the state space as the representation space of $\mathcal{X}_R$, defined as the canonical left $\mathcal{X}_R$-module $\mathfrak{V} = \mathcal{X}_R/[\sum_a \mathcal{X}_R\hat{\psi}_a^-]$. More explicitly, $\mathfrak{V}$ is the left $\mathcal{X}_R$-module generated by a vacuum state $|0\rangle$ satisfying the relation $\hat{\psi}_a^-|0\rangle = 0$ for all a, such that $\mathfrak{V} = \mathcal{X}_R|0\rangle$. Then Thm. S2.1 immediately implies that (see the comment at the end of Sec. 1 in Ref. [27]), as a vector space, $\mathfrak{V} = \mathcal{X}_R^+|0\rangle \cong \mathbb{C}_R\langle\hat{\psi}_a^+\rangle$. Furthermore, Thm. 3.2 of Ref. [27] proves that the representation $\mathfrak{V}$ of $\mathcal{X}_R$ is irreducible, and our discussion in Sec. S2 D 2 implies that it is the only irrep of $\mathcal{X}_R$ with the spectrum of n bounded from below.

In the following we find a basis for the state space $\mathfrak{V} \equiv \mathcal{X}_R^+|0\rangle$. We use the eigenvalues of the particle number operator $\hat{n}$ to decompose $\mathfrak{V}$ into a direct sum of eigenspaces of $\hat{n}$: $\mathfrak{V} = \bigoplus_{n\ge 0}\mathfrak{V}_n$. Each subspace $\mathfrak{V}_n$ is spanned by states with fixed particle number

$$\mathfrak{V}_n = \mathrm{span}\{\hat{\psi}_{a_1}^+\hat{\psi}_{a_2}^+\ldots\hat{\psi}_{a_n}^+|0\rangle | 1 \le a_j \le m, j = 1, 2, \ldots, n\}. \tag{S12}$$

Notice, however, due to the CR in Eq. (6), the states defined in the RHS of Eq. (S12) are linearly dependent. For example, the state $\hat{\psi}_a^+\hat{\psi}_b^+|0\rangle$ is the same as $\sum_{c,d} R_{ab}^{cd}\hat{\psi}_c^+\hat{\psi}_d^+|0\rangle$. A linearly independent basis for $\mathfrak{V}_n$ is established by the following theorem:

Theorem S2.2. The states $\{|n,\alpha\rangle\}_{\alpha=1}^{d_n}$ defined by Eqs. (S3-S5) (for the case $N = 1$) form a complete, linearly independent basis for $\mathfrak{V}_n$.

We now sketch the proof of Thm. S2.2. Following our discussion in the previous paragraph, the n-particle space $\mathfrak{V}_n$ of a single mode [defined in Eq. (S12)] can be identified with $\mathbb{C}_R^{(n)}\langle\hat{\psi}_a^+\rangle$, the subspace of $\mathbb{C}_R\langle\hat{\psi}_a^+\rangle$ spanned by all degree n monomials in $\hat{\psi}_a^+$s. So it remains to be proven that $\mathbb{C}_R^{(n)}\langle\hat{\psi}_a^+\rangle$ is isomorphic (as a vector space) to the space of solutions $\Psi_{a_1\ldots a_n}$ to Eq. (S3). For convenience, we define a product vector space $\mathfrak{A} \equiv \mathfrak{a}^{\otimes n}$, where $\mathfrak{a}$ is an m-dimensional vector space with basis $\{v_1, v_2, \ldots, v_m\}$. The tensor R_{cd}^{ab} defines a linear map R in the product space $\mathfrak{a}\otimes\mathfrak{a}$ as $R(v_c \otimes v_d) = \sum_{ab} R_{cd}^{ab} v_a \otimes v_b$, and this action is extended to $\mathfrak{a}^{\otimes n}$ as

$$R_{j,j+1} = \underset{(1)}{\mathbb{1}} \otimes \ldots \otimes \underset{(j-1)}{\mathbb{1}} \otimes \underset{(j,j+1)}{R} \otimes \underset{(j+2)}{\mathbb{1}} \otimes \ldots \otimes \underset{(n)}{\mathbb{1}}. \tag{S13}$$

Furthermore, we can associate a tensor $\Psi_{a_1\ldots a_n}$ to a vector in $\mathfrak{a}^{\otimes n}$ through $\Psi = \sum_{a_1\ldots a_n} \Psi_{a_1\ldots a_n} v_{a_1} \otimes v_{a_2} \otimes \ldots \otimes v_{a_n}$. Then Eq. (S3) is equivalent to

$$R_{j,j+1}\Psi = \Psi \ (\text{in } \mathfrak{a}^{\otimes n}), \ j = 1, 2, \ldots, n-1. \tag{S14}$$

In short, we need to prove that $\mathbb{C}_R^{(n)}\langle\hat{\psi}_a^+\rangle$ is isomorphic (as a vector space) to the common eigenspace (with eigenvalue +1) of all $R_{j,j+1}$. We have, as a vector space,

$$\mathbb{C}_R^{(n)}\langle\hat{\psi}_a^+\rangle \cong \frac{\mathfrak{a}^{\otimes n}}{\sum_{j=1}^{n-1}[(\mathbb{1} - R_{j,j+1})\mathfrak{a}^{\otimes n}]}, \tag{S15}$$

since $\mathbb{C}_R\langle\hat{\psi}_a^+\rangle$ is, by definition, isomorphic to the quotient of the tensor algebra $T(\mathfrak{a})$ over the quadratic relations $R(\mathfrak{a}\otimes\mathfrak{a}) = (\mathfrak{a}\otimes\mathfrak{a})$ [28], where $\mathfrak{a}$ is an m-dimensional vector space. Note that in Eq. (S15), $(\mathbb{1} - R_{j,j+1})\mathfrak{a}^{\otimes n}$ for each j is considered as a subspace of $\mathfrak{a}^{\otimes n}$, and $\sum_{j=1}^{n-1}$ means the sum of these subspaces. We now prove the following lemma:

Lemma S2.3. Let $H_1, H_2, \ldots, H_k$ be Hermitian matrices acting on a Hilbert space V. Then

$$\begin{aligned} &\mathrm{span}\{|\psi\rangle \in V \mid H_j|\psi\rangle = |\psi\rangle, 1 \le j \le k\} \\ &\cong \frac{V}{\sum_{j=1}^{k}[(\mathbb{1} - H_j)V]}. \end{aligned} \tag{S16}$$

Proof. For a Hilbert space V, and a subspace $V_1 \subseteq V$, the quotient space V/V_1 is isomorphic to the orthogonal

complement $V_1^\perp$. Therefore we have

$$\frac{V}{\sum_{j=1}^{k}[(\mathbb{1}-H_j)V]} \cong \left\{\sum_{j=1}^{k}[(\mathbb{1}-H_j)V]\right\}^\perp = \bigcap_{j=1}^{k}[(\mathbb{1}-H_j)V]^\perp\,, \qquad \text{(S17)}$$

where in the second line we used $(V_1+V_2)^\perp = V_1^\perp \cap V_2^\perp$. But since H_j is assumed to be Hermitian, $[(\mathbb{1}-H_j)V]^\perp$ is simply the eigenspace of H_j with eigenvalue $+1$, since for any $|u\rangle \in [(\mathbb{1}-H_j)V]^\perp$, we have, by definition, $\langle v|(1-H_j)|u\rangle = 0$, $\forall\, |v\rangle \in V$, implying $(1-H_j)\,|u\rangle$=0. Therefore second line of Eq. (S17) is the same as the LHS of Eq. (S16). □

Although the R-matrices in our models are not always Hermitian, there always exists a Hermitian inner product on the space $\mathfrak{A} = \mathfrak{a}^{\otimes n}$ with respect to which the matrices $\{R_{j,j+1}\}_{j=1}^{n-1}$ are all Hermitian. This is because any finite-dimensional representation of a finite group is isomorphic to a unitary representation (Theorem 4.6.2 in Ref. [29]), and in our case the matrices $\{R_{j,j+1}\}_{j=1}^{n-1}$ generate the finite group S_n (notice that if $R_{j,j+1}$ is unitary then it is Hermitian since $R_{j,j+1}^2 = \mathbb{1}$). Therefore, Lemma S2.3 still applies, implying that the RHS of Eq. (S15) is isomorphic to the common eigenspaces of $\{R_{j,j+1}\}_{j=1}^{n-1}$ defined by Eq. (S3). This concludes the proof of Thm. S2.2.

4. Many particle state space

We now prove that the states defined in Eqs. (S3-S5) form a linearly independent basis for the many particle state space $\mathcal{X}_{R,N}^{+}|0\rangle$ for any positive integer N. We need the following lemma:

Lemma S2.4. There is a vector space isomorphism $\mathcal{X}_{R,N} \cong \mathcal{X}_R^{\otimes N}$. In particular, $\mathcal{X}_R$ is isomorphic to the subalgebra of $\mathcal{X}_{R,N}$ generated by $\{\hat{\psi}_{i,a}^{\pm}|1 \le a \le m\}$, for any $a \in \{1, 2, \ldots, N\}$.

Proof. By Thm. S2.1, we have (as vector spaces) $\mathcal{X}_R \cong \mathbb{C}_R\langle\hat{\psi}_a^{+}\rangle \otimes \mathbb{C}_R\langle\hat{\psi}_a^{-}\rangle$, and $\mathcal{X}_{R,N} \cong \mathbb{C}_{\Pi\boxtimes R}\langle\hat{\psi}_a^{+}\rangle \otimes \mathbb{C}_{\Pi\boxtimes R}\langle\hat{\psi}_a^{-}\rangle$, so we only need to prove that (as a vector space) $\mathbb{C}_{\Pi\boxtimes R}\langle\hat{\psi}_a^{+}\rangle \cong \mathbb{C}_R\langle\hat{\psi}_a^{+}\rangle^{\otimes N}$. This can be proven by induction on N, where the induction step $N \to N+1$ can be proven in a similar way as Thm. S2.1. Alternatively, it is straightforward to show that $h_{\Pi\boxtimes R}(x) = h_R(x)^N$, and since for every R-matrix, $\dim \mathbb{C}_R\langle\hat{\psi}_a^{+}\rangle = h_R(1)$, we have $\dim \mathbb{C}_{\Pi\boxtimes R}\langle\hat{\psi}_a^{+}\rangle = h_R(1)^N = \dim \mathbb{C}_R\langle\hat{\psi}_a^{+}\rangle^{\otimes N}$, so as a vector space, $\mathbb{C}_{\Pi\boxtimes R}\langle\hat{\psi}_a^{+}\rangle \cong \mathbb{C}_R\langle\hat{\psi}_a^{+}\rangle^{\otimes N}$. □

While Lemma S2.4 seems natural, the non-trivial part is that the CRs (6) involving any other modes $\hat{\psi}_{j,a}^{\pm}$ (with $j \neq i$) do not give rise to any additional algebraic relations on $\{\hat{\psi}_{i,a}^{\pm}|1 \le a \le m\}$. This is a rigorous justification that different modes are mutually independent. Lemma S2.4 along with Thm. S2.2 immediately imply that the states defined in Eqs. (S3-S5) form a linearly independent basis for $\mathcal{X}_{R,N}^{+}|0\rangle$.

5. Proof of unitarity for theories based on non-unitary R-matrices

We now prove our claim in the main text that even with a non-unitary R-matrix, we can consistently define the Hermitian conjugate † on the states and operators such that $\hat{e}_{ij}^\dagger = \hat{e}_{ji}$, for $1 \le i, j \le N$, which guarantees Hermiticity of Hamiltonians and unitarity of quantum time evolution. To define the Hermitian conjugate † of operators, we need to define a Hermitian inner product $\langle \ldots | \ldots \rangle$ on the state space, and then the Hermitian conjugate of an operator $\hat{O}$ is defined as $\langle\Psi|\hat{O}^\dagger\Phi\rangle \equiv \langle\hat{O}\Psi|\Phi\rangle$, for any states $|\Psi\rangle, |\Phi\rangle$. In the following we first show that such an inner product can be consistently defined on the state space such that the induced Hermitian conjugate † satisfies $\hat{e}_{ij}^\dagger = \hat{e}_{ji}$, $\forall i, j$, and then give a more explicit definition of this inner product.

To begin, we first notice that, with the CRs in Eq. (9), the set of operators $\{\hat{e}_{ij} + \hat{e}_{ji}, i(\hat{e}_{ij} - \hat{e}_{ji})|1 \le i, j \le N\}$ spans a closed Lie algebra $\mathfrak{u}_N \cong \mathfrak{su}_N \oplus \mathfrak{u}_1$ (over the field of real numbers $\mathbb{R}$), where the $\mathfrak{u}_1$ part is $n \equiv \sum_{i=1}^{N} \hat{e}_{ii}$, and the $\mathfrak{su}_N$ part is spanned by $\{\hat{e}_{ij} + \hat{e}_{ji}, i(\hat{e}_{ij} - \hat{e}_{ji})|1 \le i < j \le N\}$ along with $\{\hat{e}_{ii} - \hat{e}_{i+1,i+1}|1 \le i \le N-1\}$. We now invoke the following theorem whose proof can be found in Ref. [30]

Theorem S2.5. For each representation ρ of a compact semisimple real Lie algebra $\mathfrak{g}$ on a finite dimensional $\mathbb{C}$-vector space V, there exists a Hermitian inner product on V such that all $\rho(x)(x \in \mathfrak{g})$ are Hermitian.

Since $\mathfrak{su}_N$ is a compact semisimple real Lie algebra, Thm. S2.5 guarantees the existence of a Hermitian inner product such that $\{\hat{e}_{ij} + \hat{e}_{ji}, i(\hat{e}_{ij} - \hat{e}_{ji})|1 \le i < j \le N\}$ and $\{\hat{e}_{ii} - \hat{e}_{i+1,i+1}|1 \le i \le N-1\}$ are all Hermitian, as long as the state space is finite dimensional. But even if the state space is infinite dimensional, we will see later that the full state space can always be decomposed as a direct sum of finite dimensional irreducible representations (irreps) of $\mathfrak{su}_N$, and Thm. S2.5 still applies to each irrep. As for the $\mathfrak{u}_1$ part, since n is proportional to the identity operator in each irrep, with the proportionality constant being the total particle number, it follows that n is also Hermitian. Since Thm. S2.5 implies that $\hat{e}_{ij} + \hat{e}_{ji} = \hat{e}_{ij}^\dagger + \hat{e}_{ji}^\dagger$ and $i(\hat{e}_{ij} - \hat{e}_{ji}) = -i(\hat{e}_{ij}^\dagger - \hat{e}_{ji}^\dagger)$, it follows that $\hat{e}_{ij}^\dagger = \hat{e}_{ji}$, for all $1 \le i, j \le N$.

The Hermitian inner product on the state space can be defined more explicitly as follows. The state space constructed in Eqs. (S3-S5) decomposes into a direct sum of different particle number sectors, and $\hat{n}$ is proportional to

identity in each sector. Each particle number sector further decomposes into a direct sum of irreps of $\mathfrak{su}_N$. We set $\langle\Psi|\Phi\rangle = 0$ if $|\Psi\rangle, |\Phi\rangle$ lie in different irreps (i.e., inequivalent irreps or different copies of equivalent irreps) of $\mathfrak{su}_N$. In this way, n is automatically Hermitian (indeed, it is real and diagonal) and the problem reduces to defining $\langle\ldots|\ldots\rangle$ within each irrep of $\mathfrak{su}_N$.

We now show that within each irrep, the inner product between any two states is uniquely determined (up to a multiplicative factor) by the requirement $\hat{e}_{ij}^\dagger = \hat{e}_{ji}$, $\forall i, j$. We show this in the framework of highest weight theory [31]. For every finite-dimensional irrep V of a finite-dimensional semisimple Lie algebra $\mathfrak{g}$, there exists a unique (up to a multiplicative constant) highest weight vector $|\Lambda\rangle$ (a $|\Lambda\rangle$ that is annihilated by all positive root operators $\hat{e}_\alpha|\Lambda\rangle = 0$), and all other weight vectors $|\Lambda'\rangle$ in V can be constructed by applying negative root operators on $|\Lambda\rangle$, i.e. $|\Lambda'\rangle = \prod_\alpha \hat{e}_{-\alpha}|\Lambda\rangle$, where the product is over some ordered set of positive roots. For the case of $\mathfrak{su}_N$, positive root operators are $\{\hat{e}_{ij}|1 \le i < j \le N\}$, negative root operators are $\{\hat{e}_{ji}|1 \le i < j \le N\}$, while $\{\hat{e}_{ii} - \hat{e}_{i+1,i+1}|1 \le i \le N-1\}$ spans the Cartan subalgebra. Without loss of generality we can assume $\langle\Lambda|\Lambda\rangle = 1$. Then for any two weight vectors $|\Lambda_1\rangle, |\Lambda_2\rangle \in V$, their inner product can be calculated as

$$\begin{aligned}\langle\Lambda_1|\Lambda_2\rangle &= \prod_{\alpha,\beta}\langle\Lambda|\hat{e}_{-\beta}^\dagger \hat{e}_{-\alpha}|\Lambda\rangle \\ &= \langle\Lambda|\prod_{\alpha,\beta}\hat{e}_\beta \hat{e}_{-\alpha}|\Lambda\rangle, \end{aligned} \tag{S18}$$

and the last line can be calculated by using the CRs between $\hat{e}_\beta$ and $\hat{e}_{-\alpha}$ (to move all the positive root operators $\hat{e}_\beta$ to the right). Notice that there may be several different ways to represent $|\Lambda_{1,2}\rangle$ in the form $\prod_\alpha \hat{e}_{-\alpha}|\Lambda\rangle$, and consequently there are different ways to compute the same inner product $\langle\Lambda_1|\Lambda_2\rangle$. Thm. S2.5 guarantees that all the different ways of computing $\langle\Lambda_1|\Lambda_2\rangle$ give the same result.

S3. RELATION BETWEEN THE SECOND AND THE FIRST QUANTIZATION FORMULATION

In this section we show the relation between the second quantized formulation of parastatistics and the first quantized wavefunction formulation we discussed in the introduction of the main text. To this end we first show the relation between the R-matrix R_{cd}^{ab} and the coefficients $(R_j)_J^I$ appearing in Eq. (3). Let the index I (and similarly for J) be a collection of n auxiliary indices $I = (a_1, a_2, \ldots, a_n)$ labeling the basis states of a product vector space $\mathfrak{A} \equiv \mathfrak{a}^{\otimes n}$ (the internal space of wavefunctions), where the basis of $\mathfrak{a}$ is $\{v_1, v_2, \ldots, v_m\}$. Now let $(R_j)_J^I$ be the matrix element of the linear mapping $R_{j,j+1}$ defined in Eq. (S13). With this choice of R_j, Eq. (3) becomes (take $n = 3$ and $j = 1$ for example)

$$\Psi^{a_1a_2a_3}(x_2, x_1, x_3) = \sum_{b_1,b_2} R_{b_1b_2}^{a_1a_2}\Psi^{b_1b_2a_3}(x_1, x_2, x_3). \tag{S19}$$

Then all the relations in Eq. (4) reduce to Eq. (5). An isomorphism between the space of n-particle wavefunctions in the first quantization formulation and the subspace of n-particle states in the second quantization formulation is defined as follows: each n-particle wavefunction $\Psi^I(x_1, \ldots, x_n)$ satisfying Eq. (3) [with the R-matrix in Eq. (S13)] corresponds to the n-particle state

$$|\Psi\rangle = \frac{1}{\sqrt{n!}}\sum_{I,x_1,\ldots,x_n}\Psi^I(x_1, \ldots, x_n)\hat{\psi}_{x_1,a_1}^+ \ldots \hat{\psi}_{x_n,a_n}^+|0\rangle, \tag{S20}$$

That Eq. (S20) indeed defines an isomorphism between the two vector spaces can be seen as follows. Note that Eq. (S3) and Eq. (S4) (for the case $N = 1$) with the R-matrix $\Pi\boxtimes R$ is the same as Eq. (3) and Eq. (S20) with the R-matrix R, respectively. Then Thm. S2.2 applied to the algebra $\mathcal{X}_{\Pi\boxtimes R} \cong \mathcal{X}_{R,N}$ shows that a linearly independent basis for the space of n-particle wavefunctions satisfying Eq. (3) correspond to a linearly independent basis for the n-particle subspace $\mathfrak{V}_n$ of the Fock space $\mathcal{X}_{\Pi\boxtimes R}^+|0\rangle \cong \mathcal{X}_{R,N}^+|0\rangle$ via the relation Eq. (S20), thereby establishing the isomorphism.

The problem with the first quantization formulation is that it is very hard to guarantee locality in this formulation, and without locality such theories are hard to be realized as elementary particles or as emergent quasiparticle excitations in locally interacting systems [32]. Part of the difficulty comes from the fact that locality puts stringent restrictions on the representation of S_n realized by $\{R_j\}_{j=1}^{n-1}$. For example, a necessary condition for locality is the cluster law introduced in some earlier works [33, 34] (their first quantization formulation of parastatistics is slightly different from ours presented in the main text but closely related, and the cluster law applies to our formulation as well). This implies that only some very special choice of the matrices $\{R_j\}_{j=1}^{n-1}$ in Eq. (3) leads to a local quantum theory, for example, the one given by Eq. (S13).

S4. DETAILS ON THE 1D SPIN MODEL AND THE MPO JWT

In this section we provide mathematical details on the 1D spin model defined in Eqs. (17-19), including (Sec. S4 A) an explicit definition of the local spin operators $\{\hat{x}_{i,a}^\pm, \hat{y}_{i,a}^\pm\}_{a=1}^m$ and (Sec. S4 B) a tensor network proof of the key properties of the MPO JWT in Eq. (18).

A. Model definition for an arbitrary R-matrix

We first define the local spin operators $\{\hat{x}^{\pm}_{i,a}, \hat{y}^{\pm}_{i,a}\}_{a=1}^{m}$ that appear in the Hamiltonian in Eq. (17), for any given R-matrix. The Hilbert space of the whole system with N sites in total is $\mathfrak{V}^{\otimes N}$, where $\mathfrak{V}$ is the Hilbert space for a single site. For any fixed $i = 1, 2, \ldots, N$, the operators $\{\hat{x}^{\pm}_{i,a}, \hat{y}^{\pm}_{i,a}\}_{a=1}^{m}$ act locally on the i-th factor space, and they are constructed to satisfy the following algebraic relations (from now on we omit the site label i)

$$\begin{aligned}
\hat{y}^-_a \hat{y}^+_b &= \sum_{c,d} R^{ac}_{bd} \hat{y}^+_c \hat{y}^-_d + \delta_{ab}, \\
\hat{y}^+_a \hat{y}^+_b &= \sum_{c,d} R^{cd}_{ab} \hat{y}^+_c \hat{y}^+_d, \\
\hat{y}^-_a \hat{y}^-_b &= \sum_{c,d} R^{ba}_{dc} \hat{y}^-_c \hat{y}^-_d, \\
\hat{x}^-_a \hat{x}^+_b &= \sum_{c,d} R^{ca}_{db} \hat{x}^+_c \hat{x}^-_d + \delta_{ab}, \\
\hat{x}^+_a \hat{x}^+_b &= \sum_{c,d} R^{dc}_{ba} \hat{x}^+_c \hat{x}^+_d, \\
\hat{x}^-_a \hat{x}^-_b &= \sum_{c,d} R^{ab}_{cd} \hat{x}^-_c \hat{x}^-_d, \\
[\hat{x}^+_a, \hat{y}^+_b] &= [\hat{x}^-_a, \hat{y}^-_b] = 0, \\
\sum_a \hat{x}^+_a \hat{x}^-_a &= \sum_a \hat{y}^+_a \hat{y}^-_a,
\end{aligned} \tag{S21}$$

While these CRs superficially resemble the CRs between paraparticle operators in Eq. (6), the difference is that the spin operators here are strictly local in that they commute on different sites, and therefore are in principle realizable, while the paraparticle operators are generally non-local operators. The first 6 lines in Eq. (S21) are shown graphically in Fig. S1.

We now define a local Hilbert space and a matrix representation of these local spin operators. The single site Hilbert space $\mathfrak{V}$ is spanned by $\{|n,\alpha\rangle\, | 1 \le \alpha \le d_n, n \in \mathbb{Z}_{\ge 0}\}$, where $\{d_n\}_{n\ge 0}$ are the same numbers that define the generalized exclusion statistics introduced in the main text, and $|n,\alpha\rangle$ is defined as

$$|n,\alpha\rangle \equiv \frac{1}{\sqrt{n!}} \sum_{a_1 a_2 \ldots a_n} \Psi^{\alpha}_{a_1 a_2 \ldots a_n} \hat{y}^+_{a_1} \hat{y}^+_{a_2} \ldots \hat{y}^+_{a_n} |0\rangle, \tag{S22}$$

where $\{\Psi^{\alpha}_{a_1 a_2 \ldots a_n}\}_{\alpha=1}^{d_n}$ is a complete set of linearly independent solutions to the system of linear equations (S3). The matrix elements of $\hat{x}^{\pm}_a, \hat{y}^{\pm}_a$ in this basis are defined as

$$\begin{aligned}
\hat{y}^{\pm}_a |n,\alpha\rangle &= \sum_{\beta=1}^{d_{n\pm 1}} Y^{\pm}_{a,\beta\alpha} |n \pm 1, \beta\rangle, \\
\hat{x}^{\pm}_a |n,\alpha\rangle &= \sum_{\beta=1}^{d_{n\pm 1}} X^{\pm}_{a,\beta\alpha} |n \pm 1, \beta\rangle,
\end{aligned} \tag{S23}$$

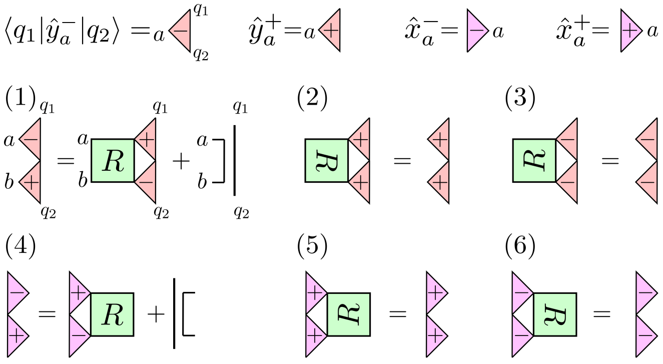

FIG. S1. Graphical representation of the CRs between the local spin operators $\{\hat{x}^{\pm}_a, \hat{y}^{\pm}_a\}_{a=1}^{m}$ in Eq. (S21). The matrix elements of each operator $\{\hat{x}^{\pm}_a, \hat{y}^{\pm}_a\}_{a=1}^{m}$ is a tensor (represented by the triangles) with two quantum indices (e.g. the indices q_1 and q_2 shown in figure) and one auxiliary index (e.g. the index a), and the R-matrix (represented by a square) is a tensor with four auxiliary indices. Matrix multiplication goes from top to bottom in the quantum space and from left to right in the auxiliary space.

where the coefficients $Y^{\pm}_{a,\beta\alpha}, X^{\pm}_{a,\beta\alpha}$ are given by

$$\begin{aligned}
\sum_\beta Y^+_{a,\beta\alpha} \Psi^{\beta}_{a_0 a_1 \ldots a_n} &= \frac{1}{\sqrt{n+1}} \bar{Y}^{a_0 a_1 \ldots a_n}_{a\ b_1 \ldots b_n} \Psi^{\alpha}_{b_1 \ldots b_n}, \\
\sum_\beta X^+_{a,\beta\alpha} \Psi^{\beta}_{a_n \ldots a_1 a_0} &= \frac{1}{\sqrt{n+1}} \bar{X}^{a_n \ldots a_1 a_0}_{b_n \ldots b_1 a} \Psi^{\alpha}_{b_n \ldots b_1}, \\
\sum_\beta Y^-_{a,\beta\alpha} \Psi^{\beta}_{a_2 \ldots a_n} &= \sqrt{n} \Psi^{\alpha}_{a a_2 \ldots a_n}, \\
\sum_\beta X^-_{a,\beta\alpha} \Psi^{\beta}_{a_n \ldots a_2} &= \sqrt{n} \Psi^{\alpha}_{a_n \ldots a_2 a},
\end{aligned} \tag{S24}$$

where the tensors $\bar{Y}, \bar{X}$ are defined as

$$\begin{aligned}
\bar{Y}_{01\ldots n} &= 1 + R_{01} + R_{12}R_{01} + \ldots + R_{n-1,n} \cdots R_{12} R_{01}, \\
\bar{X}_{n\ldots 10} &= 1 + R_{10} + R_{21}R_{10} + \ldots + R_{n,n-1} \cdots R_{21} R_{10},
\end{aligned} \tag{S25}$$

where we use the tensor notation introduced in Eqs. (S13,S14). It is straightforward to check that $\hat{x}^{\pm}_a, \hat{y}^{\pm}_a$ defined this way satisfy all the CRs in Eq. (S21), and Eq. (S23) is consistent with Eq. (S22). In addition, we have the relation

$$[\hat{n}, \hat{x}^{\pm}_a] = \pm \hat{x}^{\pm}_a, \quad [\hat{n}, \hat{y}^{\pm}_a] = \pm \hat{y}^{\pm}_a, \tag{S26}$$

where $\hat{n} \equiv \sum_a \hat{x}^+_a \hat{x}^-_a = \sum_a \hat{y}^+_a \hat{y}^-_a$.

We can now compute $\hat{T}^{\pm}_{ab}$ using their definition $\hat{T}^{\pm}_{ab} \equiv \mp[\hat{y}^{\pm}_a, \hat{x}^{\mp}_b]$, and the matrix elements of $\hat{x}^{\pm}_a, \hat{y}^{\pm}_a$ in Eq. (S24). The matrix elements of $\hat{T}^{\pm}_{ab}$ are defined similarly as

$$\hat{T}^{\pm}_{ab} |n,\alpha\rangle = \sum_{\beta=1}^{d_n} T^{\pm}_{ab,\beta\alpha} |n,\beta\rangle, \tag{S27}$$

where the coefficients $T^{\pm}_{ab,\beta\alpha}$ are

$$\sum_{\beta} T^{+}_{ab,\beta\alpha}\Psi^{\beta}_{a_1 a_2\ldots a_n} = \sum_{a'_1,\ldots,a'_n} \Psi^{\alpha}_{a'_1,a'_2\ldots,a'_n}$$

$\times$ 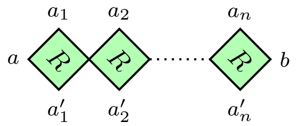 ,

$$\sum_{\beta} T^{-}_{ab,\beta\alpha}\Psi^{\beta}_{a_1 a_2\ldots a_n} = \sum_{a'_1,\ldots,a'_n} \Psi^{\alpha}_{a'_1 a'_2\ldots a'_n} \quad \text{(S28)}$$

$\times$ 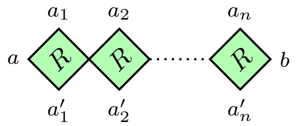 .

All the results above are valid for an arbitrary R-matrix satisfying Eq. (5) of the main text, including non-unitary ones. When the R-matrix is unitary, we require that $\{\Psi^{\alpha}_{a_1 a_2\ldots a_n}\}^{d_n}_{\alpha=1}$ is normalized as in Eq. (S6), and we define an inner product on the on-site Hilbert space $\langle n', \beta | n, \alpha\rangle = \delta_{n'n}\delta_{\alpha\beta}$. Then one can check that the matrix representation of $\{\hat{x}^{\pm}_{i,a}, \hat{y}^{\pm}_{i,a}\}^{m}_{a=1}$ defined in Eq. (S23) satisfies $\hat{x}^{+}_{a} = (\hat{x}^{-}_{a})^{\dagger}$, $\hat{y}^{+}_{a} = (\hat{y}^{-}_{a})^{\dagger}$. Therefore the 1D spin model is Hermitian. Furthermore, we have $\hat{T}^{+}_{ab} = (\hat{T}^{-}_{ab})^{\dagger}$, leading to $\hat{\psi}^{+}_{i,a} = (\hat{\psi}^{-}_{i,a})^{\dagger}$, where $\hat{\psi}^{+}_{i,a}$ are defined by the MPO JWT in Eq. (18). In a similar way, one can show that the 2D model defined in Eq. (32) is also Hermitian when R is unitary. Unitary R-matrices have been classified up to a certain equivalence relation in Ref. [35], their Hilbert series all have the form in Eq. (5.7) of Ref. [35].

We can give the matrix elements of $\{\hat{x}^{\pm}_{a}, \hat{y}^{\pm}_{a}\}^{m}_{a=1}$ more explicitly for the R-matrices given in Tab. I. One finds that for the R-matrices in Ex. 1 and Ex. 2, the corresponding spin models are free fermion solvable models that fall into the classification in Ref. [36]. In particular, Ex. 2 corresponds to m decoupled chains of 1D XY models, each of which maps to a 1D free fermion chain. The spin model for the R-matrix in Ex. 3 has been presented in the main text. For the R-matrix in Ex. 4, the local Hilbert space $\mathfrak{V}$ is $m+2$-dimensional, with basis states $|0\rangle, \{|1,b\rangle\}^{m}_{b=1}, |2\rangle$; the non-zero matrix elements of $\hat{y}^{\pm}_{a}$ are $\hat{y}^{+}_{a}|0\rangle = |1,a\rangle$, $\hat{y}^{-}_{a}|1,b\rangle = \delta_{ab}|0\rangle$, $\hat{y}^{+}_{a}|1,b\rangle = \xi_{ab}|2\rangle$, and $\hat{y}^{-}_{a}|2\rangle = \sum_b \lambda_{ab}|1,b\rangle$; and the non-zero matrix elements of $\hat{x}^{\pm}_{a}$ are $\hat{x}^{+}_{a}|0\rangle = |1,a\rangle$, $\hat{x}^{-}_{a}|1,b\rangle = \delta_{ab}|0\rangle$, $\hat{x}^{+}_{a}|1,b\rangle = \xi_{ba}|2\rangle$, and $\hat{x}^{-}_{a}|2\rangle = \sum_b \lambda_{ba}|1,b\rangle$. The action of the operators $\hat{x}^{\pm}_{i}, \hat{y}^{\pm}_{i}$ and n on the orthonormal basis states are shown in Fig. S2.

The spin model Hamiltonian in Eq. (17) is not Hermitian for the non-unitary R-matrix in Ex. 4 for $m \geq 3$ [37], since $\hat{x}^{+}_{a} \neq (\hat{x}^{-}_{a})^{\dagger}, \hat{y}^{+}_{a} \neq (\hat{y}^{-}_{a})^{\dagger}$. However, $\hat{H}$ is parity-time symmetric [38, 39], and all its eigenvalues are real. To be precise, let P be the parity operator that generates the chain reflection symmetry, and let T be the time-reversal symmetry, which, in our spin model, is simply complex conjugation. Using the explicit representation of the matrices λ_{ab}, ξ_{ab} in Tab. I, we see that

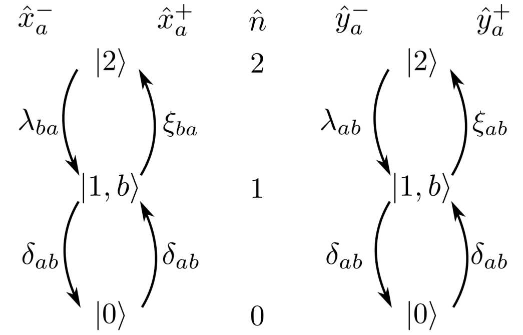

FIG. S2. The action of the operators $\hat{x}^{\pm}_{a}, \hat{y}^{\pm}_{a}$ and $\hat{n}$ on the basis states $|0\rangle$, $\{|1,b\rangle\}^{m}_{b=1}$, $|2\rangle$, for the spin model corresponding to the R-matrix in Ex. 4.

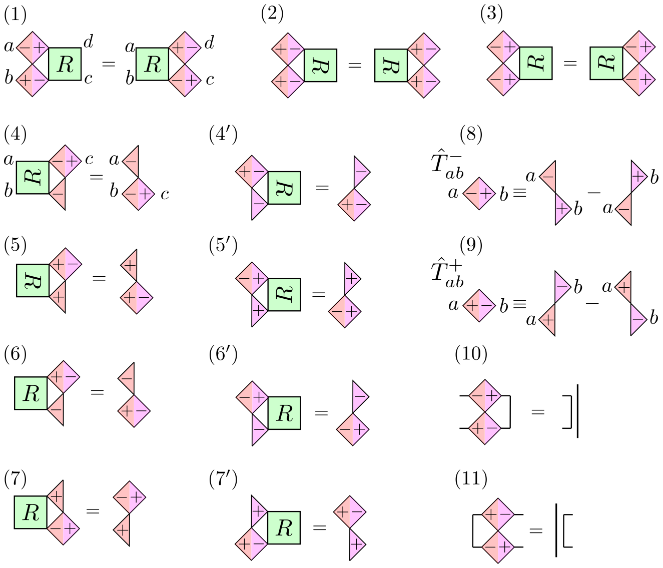

FIG. S3. Graphical representation of the CRs between the local spin operators $\hat{T}^{\pm}_{ab}$ and $\{\hat{x}^{\pm}_{a}, \hat{y}^{\pm}_{a}\}^{m}_{a=1}$.

$\lambda^{*}_{ab} = \lambda_{ba}, \xi^{*}_{ab} = \xi_{ba}$, and therefore, the time-reversal operation T simply swaps the operators $\hat{x}^{\pm}_{a} \leftrightarrow \hat{y}^{\pm}_{a}$ in the Hamiltonian $\hat{H}$, which can subsequently be undone by P. Thus, $\hat{H}$ is invariant under the combined operation PT. As we already see from the exact solution of the spectrum, all eigenvalues of $\hat{H}$ are real. This kind of PT-symmetric Hamiltonian can still define unitary quantum dynamics [38], so it is interesting to conceive an experimental platform that realizes the non-Hermitian Hamiltonian in Eq. (17) with this kind of emergent paraparticle. It is also interesting to investigate if the parastatistics in Ex. 4 can alternatively be realized in a Hermitian spin model.

B. Generalized Jordan-Wigner transformations

We now prove that the emergent paraparticles' creation and annihilation operators defined by the MPO

JWT in Eq. (18) do satisfy the parastatistical CRs in Eq. (6), and the spin Hamiltonian in Eq. (17) is mapped to the free paraparticle Hamiltonian in Eq. (19). An important first step is to prove the algebraic relations between the local spin operators $\hat{T}^{\pm}_{ab}$ and $\{\hat{x}^{\pm}_a, \hat{y}^{\pm}_a\}_{a=1}^m$ as shown graphically in Fig. S3.

1. *Proof of CRs in Fig. S3*

Indeed, all the CRs in Fig. S3 can be proved by straightforward computations using the explicit definition of $\hat{x}^{\pm}_a, \hat{y}^{\pm}_a$ and $\hat{T}^{\pm}_{ab}$ given above. A smarter proof strategy is given below.

First, we use Fig. S3.8 and Fig. S3.9 as the definitions of the tensors $T^- =$ 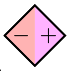 and $T^+ =$ 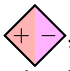, respectively. Then Fig. S3.7 can be proved easily using Fig. S2.2 and $[\hat{x}^+_a, \hat{y}^+_b] = 0$, and Fig. S3.7' is proved similarly. Then Fig. S3.5 is proved as follow. Denote this equation as $\hat{l}_A = \hat{r}_A$, where A is a collective label for all the open indices. Notice that
(1). This equation holds when acting on $|0\rangle$: $\hat{l}_A |0\rangle = \hat{r}_A |0\rangle$;
(2). Both sides of this equation transform in the same way when we commute them with $\hat{x}^+_a$, i.e.

$$\hat{l}_A \hat{x}^+_a = \sum_{B,b} w^{Bb}_{Aa} \hat{x}^+_b \hat{l}_B,$$
$$\hat{r}_A \hat{x}^+_a = \sum_{B,b} w^{Bb}_{Aa} \hat{x}^+_b \hat{r}_B, \tag{S29}$$

where w^{Bb}_{Aa} are some constant coefficients.
We can therefore conclude that $\hat{l}_A = \hat{r}_A$ in the whole space since the whole space is spanned by states of the form $\hat{x}^+_{a_1} \ldots \hat{x}^+_{a_n} |0\rangle$. Fig. S3.5' is proved in the same way, the only difference is that in step (2) above we use the fact that both sides transform in the same way when we commute them with $\hat{y}^+_a$, since Fig. S3.7 tells us how $\hat{T}^-$ commutes with $\hat{y}^+_a$.

Now that S3.5, S3.5', S3.7, and S3.7' are proved, we know how $T^{\pm}$ commutes with $\hat{x}^+_a, \hat{y}^+_a$. All the remaining equations in Fig. S3 can be proved in the same way using steps (1) and (2) above, i.e. by showing that the equation is true when acting on $|0\rangle$, and that both sides transform in the same way when we commute them with either $\hat{x}^+_a$ or $\hat{y}^+_a$, implying that the equation holds on the whole space.

2. *The spin-paraparticle mapping via the MPO JWT*

With all those relations shown in Fig. S3, Eq. (6) can be proven. For example, in Fig. S4 we show the proof of the first parastatistical CR in Eq. (6) for the $\hat{\psi}^{\pm}_{i,a}$ defined in terms of the spin operators in Eq. (18) and the algebraic relations in Fig. S3. Other relations in Eq. (6)

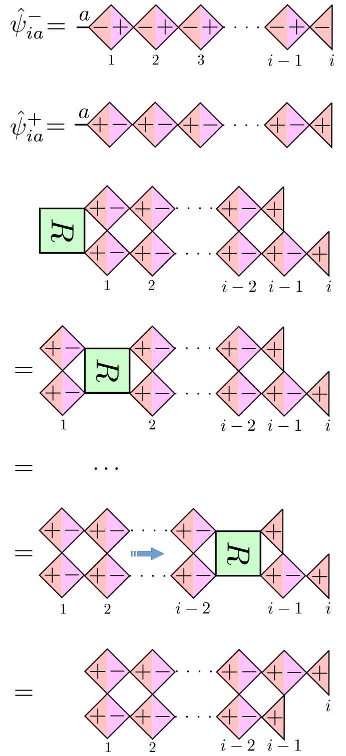

FIG. S4. The graphical proof of the first relation in Eq. (6), using the definition of $\hat{\psi}^{\pm}_{i,a}$ in Eq. (18) and the algebraic relations in Fig. S3.

are proven in a similar way. Furthermore, one can insert Eq. (18) into Eq. (19) to reproduce Eq. (17), using the last two relations in Fig. S3 and a similar graphical manipulation as in Fig. S4. This proves the exact mapping from the 1D spin model to free paraparticles.

S5. THE 2D SOLVABLE SPIN MODEL WITH EMERGENT PARAPARTICLES

In this section we provide technical details for the 2D solvable spin models with emergent free paraparticles, introduced in the Methods of the main text. Specifically, in Sec. S5 A we define the tensors $u^{\pm}_L, u^{\pm}_R, v^{\pm}_L, v^{\pm}_R$ that appear in the three body interaction terms, in Sec. S5 B we prove two important properties of the solvable spin Hamiltonian, and then in Sec. S5 C we prove important properties of the 2D MPO JWT which eventually lead to free paraparticle representation in Thm. 1. Finally, in Sec. S5 D we show that paraparticles in the 2D solvable spin model can be locally created and measured at special points on the boundary.

A. Definition of the tensors u_L, u_R, v_L, v_R

Let 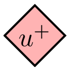 and 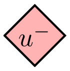 be tensors with 4 indices, where the horizontal ones are parastatistical indices (taking values $1, \dots, m$) and the vertical (taking values $1, \dots, Q$) are quantum, and suppose that they satisfy the tensor equations

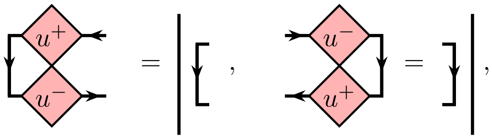

(S30)

where arrows are used to distinguish between input and output indices when we group the parastatistical and quantum indices to view u^+ and u^- as $mQ \times mQ$ matrices, and we use the convention that vertical indices always go from top to bottom by default. More precisely, with matrix notation, we have

$[u^+]^{b\beta}_{a\alpha} =$ 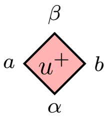 , $[u^-]^{a\alpha}_{b\beta} =$ 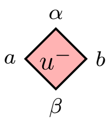 . (S31)

When we collect indices in this way, Eq. (S30) simply means that u^+ and u^- are matrix inverse of each other. We use u to collectively denote the tensors u^+, u^-. We will also use the notation $\hat{u}^+_{ab}$ to denote the quantum operator 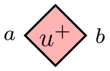 and similarly $\hat{u}^-_{ab}$ to denote the operator 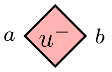 .

Definition S5.1. Let R and R' be two involutive solutions to the YBE with the same m (i.e., same dimension). We say that the tensors u, v satisfy the (R, R') commutation relation, denoted as $u \overset{RR'}{\longleftrightarrow} v$, if the following systems of relations hold:

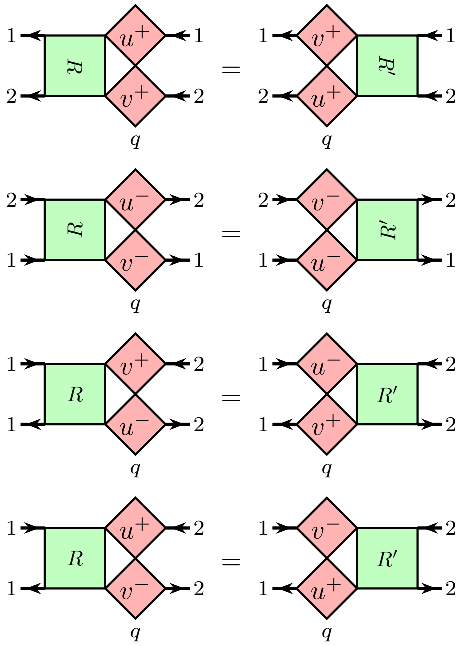

(S32)

Remark S5.1. If we view $u^\pm$ and $v^\pm$ as a $mQ \times mQ$ matrices (with index convention explained above), Eq. (S32) can be rewritten as the following matrix equations

$$
\begin{aligned}
u^+_{1q} v^+_{2q} R_{12} &= R'_{12} v^+_{1q} u^+_{2q}, \\
R_{12} u^-_{2q} v^-_{1q} &= v^-_{2q} u^-_{1q} R'_{12}, \\
v^+_{2q} R_{12} u^-_{2q} &= u^-_{1q} R'_{12} v^+_{1q}, \\
u^+_{2q} R_{12} v^-_{2q} &= v^-_{1q} R'_{12} u^+_{1q},
\end{aligned}
\tag{S33}
$$

where both sides of the equation act on a $m \times m \times Q$ dimensional product vector space $V_1 \otimes V_2 \otimes V_q$, and the subscripts indicate which factor space a tensor acts on. It is straightforward to see that the four equations in Eq. (S33) are equivalent to each other under the invertibility condition (S30). We present all four for readers' convenience, since they all are important for the 2D MPO JWT.

Remark S5.2. Recall that in defining the 1D MPO JWT we introduced the tensors $T^+ =$ 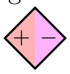 and $T^- =$ 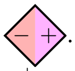. If we use the same tensor indexing convention as $u^\pm$ explained above, then their CRs shown in Fig. S3 can be succinctly rewritten as $T \overset{RR}{\longleftrightarrow} T$.

Now let u_L, v_L, u_R, v_R be invertible [i.e. Eq. (S30) holds for $u = u_L, v_L, u_R, v_R$] tensors satisfying the diagram of CRs shown in Fig. S5. Such tensors can always be constructed for any R matrix associated with a finite dimensional $\mathbb{C}^*$-triangular Hopf algebra [40–43], and in this construction Q is the dimension of the Hopf algebra. In particular, the set-theoretical R matrix defined in Eq. (29) is associated with a 64-dimensional Hopf algebra $\mathcal{H}_{64}$ [44], so in this case we have $m = 4$ and $Q = 64$.

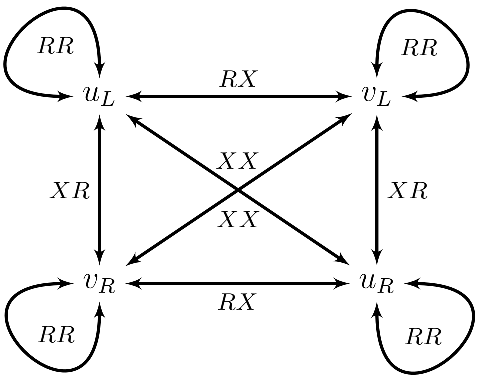

FIG. S5. The diagram of CRs satisfied by the tensors u_L, v_L, u_R, v_R. The two sided arrow $u \overset{RR'}{\longleftrightarrow} v$ means that u, v satisfy the (R, R') CR defined in Eq. (S32). A self loop on u with relation RR means $u \overset{RR}{\longleftrightarrow} u$. X is the two qudit swap gate (X has the same dimension as R).

The detailed mathematical construction is quite technical, which we omit here, but in the accompanying Mathematica code [45], we give numerical representations of these tensors [in the code, these tensors are stored as $mQ \times mQ$ matrices, using the indexing convention in Eq. (S31)], and verify that the satisfy all the CRs in Fig. S5.

Remark S5.3. Notice that Eq. (S32) is invariant under a sign flip of either the u tensor or the v tensor, therefore we have a freedom to choose the signs of the tensors u_L, v_L, u_R, v_R. Indeed, we can choose their signs independently at each white site of the 2D lattice. The mapping to free paraparticles is valid regardless of this sign choice. However, this sign choice does affect the spectrum of the resulting free paraparticle Hamiltonian $\hat{H}_2$ in Eq. (35), since the signs of the tunneling constants of the free paraparticles depend on this sign choice. In our specific model, it turns out that the tensors u_L, v_L, u_R, v_R we construct from the Hopf algebra $\mathcal{H}_{64}$ satisfy

$$v_L^+ u_L^- v_R^+ u_R^- = -1. \tag{S34}$$

which is verified in the accompanying Mathematica code [45]. If we directly use these tensors to construct the 2D solvable spin model in Eq. (32), the resulting free paraparticle Hamiltonian will have a π-flux on each square plaquette of the lattice (including all the white, gray, and colored plaquettes in Fig. 2), which brings some unnecessary inconvenience for our discussions later. We therefore use our freedom of choosing the signs of u_L, v_L, u_R, v_R and flip the signs of v_L and u_L on each horizontal triangle in Fig. 2 (a triangle is horizontal if its longest edge is horizontal; for example the v_R-triangle with vertices $0, 1, 2$ in Fig. 2 is horizontal). We use this sign convention throughout this paper: whenever we mention a u_L or v_L tensor on a horizontal triangle, we mean the sign-flipped one. Notice that this sign-flip also changes the signs of the conserved loop terms $\hat{A}_\nu$ and $\hat{B}_p$, since each of them involves exactly one of u_L, v_L. This is why in Eq. (32), we define $\hat{H}_1 = \sum_\nu \hat{A}_\nu + \sum_p \hat{B}_p$, so that with the additional sign-flip, our definition actually agrees with the definition $\hat{H} = -\sum_\nu \hat{A}_\nu - \sum_p \hat{B}_p$ in the quantum double model literature (see also the note after Fact S5.2).

B. Important properties of the solvable Hamiltonian

We begin by proving a few simple facts about the model defined in Eq. (32) of the main text.

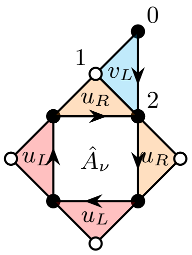

FIG. S6. Example of two operators that commute: an 8-body term $\hat{A}_\nu$ and a 3-body term v_L. (See text for proof.)

Fact S5.1. All loop terms in $\hat{H}_1$ mutually commute, and commute with each individual 3-body term in $\hat{H}_2$. Therefore, the loop terms are conserved quantities and eigenstates of $\hat{H}$ can be labeled by their common eigenvalues.

Proof. This is proved by elementary tensor network manipulations. As an example, we prove the commutativity between the red loop term $\hat{A}_\nu$ and the cyan v_L triangle term $\hat{h}_{02}$ on its top, as shown in Fig. S6. Denote by $\hat{A}_\nu = \hat{A}_\nu^+ + \hat{A}_\nu^-$ and $\hat{h}_{02} = \hat{h}_{02}^+ + \hat{h}_{02}^-$, where $\hat{A}_\nu^+$ and $\hat{h}_{02}^+$ are the terms involving u_R^+ and v_L^+, respectively, and $\hat{A}_\nu^-$ and $\hat{h}_{02}^-$ are their Hermitian conjugate. We first prove that $[\hat{A}_\nu^+, \hat{h}_{02}^+] = 0$. To this end, it is enough to prove that their actions on the overlapping sites (labeled $1, 2$ in Fig. S6) commute. We have

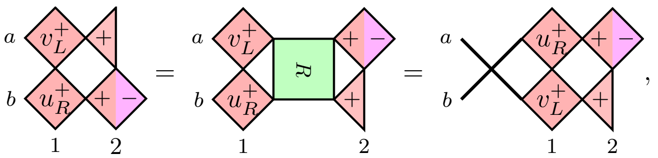

where we used the CR between u_R and v_L defined in Eq. (S32) and Fig. S5, and the CR between $\hat{y}^+$ and $\hat{T}^+$

in Fig. S3. Similarly, we have

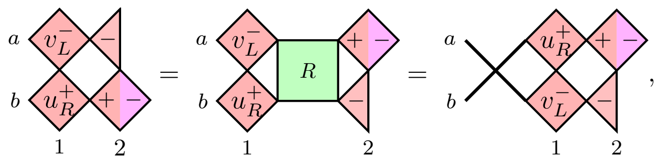

which proves that $[\hat{A}_\nu^+, \hat{h}_{02}^-] = 0$. By taking Hermitian conjugates, we obtain $[\hat{A}_\nu^-, \hat{h}_{02}^+] = [\hat{A}_\nu^-, \hat{h}_{02}^-] = 0$, therefore, $[\hat{A}_\nu, \hat{h}_{02}] = 0$. The commutativity between the loop terms and other triangle terms and between different loop terms is proved similarly. □

Fact S5.2. The 2D spin model can be viewed as a significant generalization of Kitaev's quantum double model as follows: the space of states in which all qudits on the black dots are in the state $|0\rangle$ is invariant under all individual terms in $\hat{H}_1$ and $\hat{H}_2$. In this sector, $\hat{H}_2$ vanishes and $\hat{H}_1$ reduces to Kitaev's quantum double model [46–52] constructed from a minimal $\mathbb{C}^*$-triangular Hopf algebra $\mathcal{H}_{64}$. In particular, it has a unique ground state with the open boundary condition shown in Fig. 2, where the system size L is odd (L is the total number of black sites in one direction, e.g. $L = 7$ in Fig. 2).

Proof. We have $\hat{x}_a^- |0\rangle = \hat{y}_a^- |0\rangle = 0$ and $\hat{T}_{ab}^{\pm} |0\rangle = \delta_{ab} |0\rangle$, therefore in this sector all the 3 body interactions vanish, the black dots completely decouple, and the 8 body interactions reduce to 4 body interaction between the white dots. In the accompanying code [45] we check in a small lattice with $L = 3$ that they are the vertex and plaquette terms in Kitaev's quantum double model based on the Hopf algebra $\mathcal{H}_{64}$, and that $\hat{H}_1$ has a unique ground state (these claims can all be proved mathematically, which we present in a future work).

[Note: The precise relation to Kitaev's quantum double model based on $\mathcal{H}_{64}$ is explained as follow. In our model the loop terms $\hat{A}_\nu, \hat{B}_p$ both have eigenvalues $\{-4, -2, 0, +2, +4\}$, while in the literature [46, 49] Kitaev's quantum double model is often written as sum of local projectors. However, if we replace $\hat{H}_1$ in Eq. (32) by $\hat{H}_1' = -\sum_\nu f(\hat{A}_\nu/2) - \sum_p f(\hat{B}_p/2)$, where $f(x) = (x+1)x(x-1)(x-2)/4!$ such that $f(\hat{A}_\nu/2)$ and $f(\hat{B}_p/2)$ are projectors to the lowest eigenstate of $\hat{A}_\nu$ and $\hat{B}_p$, respectively, then $\hat{H}_1'$ exactly reproduces Kitaev's quantum double model Hamiltonian in the sector where all the black sites are in the state $|0\rangle$. Since $\hat{A}_\nu$ and $\hat{B}_p$ are both conserved, and the ground state subspaces of $\hat{H}_1$ and $\hat{H}_1'$ are exactly the same, using either $\hat{H}_1$ or $\hat{H}_1'$ in Eq. (32) lead to the same conclusions. Therefore in this paper, we take the relatively simple choice $\hat{H}_1$, and all the existing knowledge about the ground state of Kitaev's quantum double model still applies to our model.] □

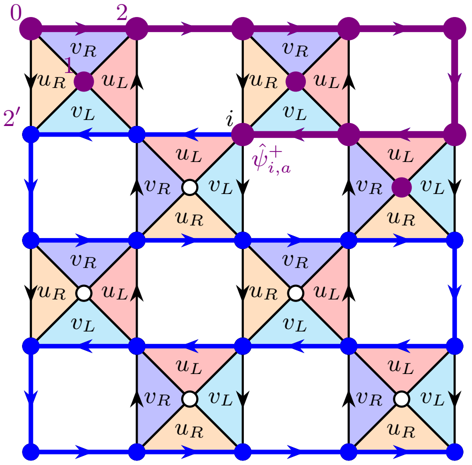

The primary path P_1

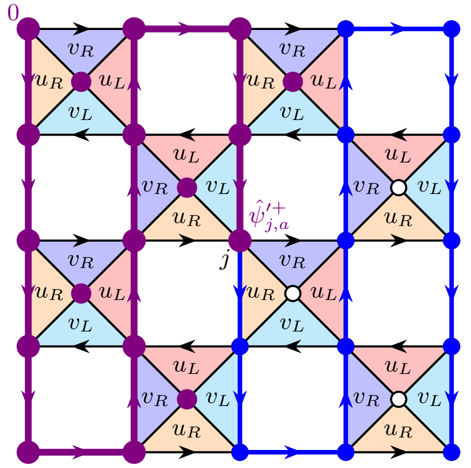

The secondary path P_2

FIG. S7. Example of a paraparticle operator defined on the primary path P_1 (top) and the secondary path P_2 (bottom). The paraparticle operator $\hat{\psi}_{i,a}^{\pm} \equiv \hat{\psi}_{i,a}^{\pm}(P_1)$ is a matrix product string operator acting on all the sites on P_1 starting at the lattice origin 0 all the way up to the site i, as well as all the auxiliary sites adjacent to the path P_1 (all sites on which $\hat{\psi}_{i,a}^{\pm}$ acts nontrivially are colored purple). The paraparticle operator $\hat{\psi}_{j,a}'^{\pm} \equiv \hat{\psi}_{j,a}^{\pm}(P_2)$ on the secondary path P_2 is defined similarly.

C. The 2D MPO JWT and the proof of Thm. 1

In the Methods we defined the paraparticle creation and annihilation operators $\hat{\psi}_{i,a}^{\pm}$ via a 2D MPO JWT of the form Eq. (34), on an arbitrary directed path in the lattice connecting the lattice origin to the site i. In this section we prove several important properties of these operators which eventually lead to Thm. 1 in the Methods, including their commutativity with the local terms in $\hat{H}_1$, and that their actions in the zero-vortex sector are path-independent and satisfy the fundamental CRs

in Eq. (6).

Consider the primary path P_1 shown in the left of Fig. S7, which travels through all the black sites of the entire lattice. We denote by $\hat{\psi}^{\pm}_{i,a} \equiv \hat{\psi}^{\pm}_{i,a}(P_1)$ the paraparticle operators acting on the substring of P_1 starting from the lattice origin 0 and ending at site i, as shown in Fig. S7, and the paraparticle operators $\hat{\psi}'^{\pm}_{j,a} \equiv \hat{\psi}^{\pm}_{j,a}(P_2)$ on the secondary path P_2 are defined similarly. The following fact can be proved in an identical way as in the 1D case

Fact S5.3. The set of all paraparticle operators $\{\hat{\psi}^{\pm}_{i,a}\}$ on P_1 satisfy the CRs in Eq. 6 of the main text. For any edge $\langle ij\rangle \in P_1$, we have

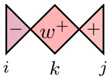

$$= \sum_{a=1}^{m} \hat{\psi}^{+}_{j,a}\hat{\psi}^{-}_{i,a},$$

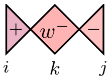

$$= \sum_{a=1}^{m} \hat{\psi}^{+}_{i,a}\hat{\psi}^{-}_{j,a}, \qquad \text{(S35)}$$

for $w = u_L, u_R, v_L$, or v_R. A similar result holds for the secondary path P_2. Moreover, on every site i, we have

$$\hat{n}_i \equiv \sum_{a=1}^{m} \hat{\psi}^{+}_{i,a}\hat{\psi}^{-}_{i,a} = \sum_{a=1}^{m} \hat{y}^{+}_{i,a}\hat{y}^{-}_{i,a} = \sum_{a=1}^{m} \hat{\psi}'^{+}_{i,a}\hat{\psi}'^{-}_{i,a}. \qquad \text{(S36)}$$

The space of states mentioned in Fact S5.2 correspond to paraparticle vacuum, since we have $\hat{\psi}^{-}_{i,a}|0\rangle = \hat{\psi}'^{-}_{i,a}|0\rangle = 0$, therefore, $\hat{n}_i|0\rangle = 0$. The following fact describes a construction of all other states in the Hilbert space:

Fact S5.4. The paraparticle operators $\{\hat{\psi}^{\pm}_{i,a}\}$ commute with all individual terms in $\hat{H}_1$. Moreover, the Hilbert space is spanned by states of the form

$$\hat{\psi}^{+}_{i_1,a_1}\hat{\psi}^{+}_{i_2,a_2}\ldots\hat{\psi}^{+}_{i_n,a_n}|\Omega\rangle, \qquad \text{(S37)}$$

where n is a non-negative integer, $i_1,\ldots,i_n$ are labels of the black sites (not necessarily distinct), $a_1,\ldots,a_n \in \{1,2,\ldots,m\}$, and $|\Omega\rangle$ has no paraparticles (is $|0\rangle$ on all black dots) and is a common eigenstate of all the loop operators.

Proof. That $\{\hat{\psi}^{\pm}_{i,a}\}$ commute with all loop terms is proved in a similar way as in Fact S5.1. Since $\{\hat{A}_\nu, \hat{B}_p, \hat{n}_i\}$ mutually commute, the full Hilbert space is spanned by their common eigenstates. For any common eigenstate $|\Psi\rangle$, let n_Ψ be the total number of paraparticles in $|\Psi\rangle$, i.e. $\hat{n}|\Psi\rangle = n_\Psi|\Psi\rangle$, where $\hat{n} = \sum_i \hat{n}_i$ is the total particle number operator. In the following we use induction on n_Ψ to prove that $|\Psi\rangle$ is a linear combination of states of the form (S37).

The induction hypothesis is trivially true for $n_\Psi = 0$. Now assume that the hypothesis is true for any common eigenstate $|\Psi\rangle$ with $n_\Psi = k$. For a common eigenstate $|\Psi\rangle$ with $n_\Psi = k+1$, let j be a black site such that $\hat{n}_j|\Psi\rangle = n_j|\Psi\rangle > 0$. Then we have

$$\begin{aligned}|\Psi\rangle &= \frac{1}{n_j}\hat{n}_j|\Psi\rangle \\ &= \frac{1}{n_j}\sum_{a=1}^{m}\hat{\psi}^{+}_{j,a}\hat{\psi}^{-}_{j,a}|\Psi\rangle. \qquad \text{(S38)}\end{aligned}$$

It is clear that each state $\hat{\psi}^{-}_{j,a}|\Psi\rangle$ is either zero, or a common eigenstate of $\{\hat{A}_\nu, \hat{B}_p, \hat{n}_i\}$ with total paraparticle number $n_\Psi - 1 = k$. By the induction hypothesis, $\hat{\psi}^{-}_{j,a}|\Psi\rangle$ is a linear combination of states of the form (S37). Then the second line of Eq. (S38) implies that $|\Psi\rangle$ is also a linear combination of states of the form (S37), which completes the induction step. Therefore, the induction hypothesis is true for any $n_\Psi \in \mathbb{Z}_{\geq 0}$. □

The next lemma shows that $\{\hat{\psi}^{+}_{i,a}\}$ and $\{\hat{\psi}'^{+}_{i,a}\}$ defined on different paths also satisfy the canonical CR in Eq. (6) of the main text:

Lemma S5.1. The paraparticle creation operators defined on different paths P_1 and P_2 satisfy the CRs

$$\hat{\psi}^{+}_{i,a}(P_1)\hat{\psi}^{+}_{j,b}(P_2) = \sum_{cd} R^{cd}_{ab}\hat{\psi}^{+}_{j,c}(P_2)\hat{\psi}^{+}_{i,d}(P_1). \qquad \text{(S39)}$$

[Indeed, Lemma S5.1 and the subsequent Lemma S5.2 hold for any two paths P_1 and P_2 in the lattice that start from the lattice origin 0, but for the purpose of proving Thm. 1 in the main text, it is enough to only consider the case when P_1 and P_2 are the primary and second paths shown in Fig. S7.]

It is not hard to be convinced about the correctness of Lemma S5.1 by considering some simple cases, as the treatment is very similar to the tensor network manipulations in the proof of Fact S5.1. Below we show the proof of a simple case where both i and j are adjacent to the lattice origin 0. The labeling of sites is shown in Fig. S7, so $i = 2$ and $j = 2'$ in this case. We have a tensor graphical derivation of Eq. (S39):

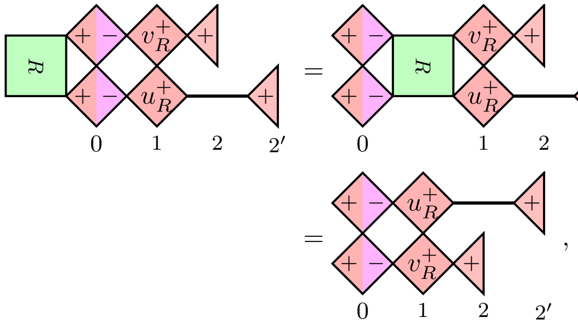

where in the first step we used $T \overset{RR}{\longleftrightarrow} T$, and in the second step we used $u_R \overset{RX}{\longleftrightarrow} v_R$. The full proof of Lemma S5.1 that takes into account all possible cases is quite tedious: one begins by proving certain CRs satisfied by the MPO JW strings of $\hat{\psi}^{+}_{i,a}$ using induction on

the string length, where the base case is given by the CRs of u_L, u_R, v_L, v_R (which are essentially length-1 strings) in Fig. S5, then one uses the CRs of the JW strings along with the CRs in Fig. S3 to prove Eq. (S39), in a way similar to the graphical proof of the 1D case shown in Fig S4 and the proof of the simple case shown above. We omit the full proof in this paper.

The following lemma proves that the actions of $\hat{\psi}^{\pm}_{i,a}$ and $\hat{\psi}'^{\pm}_{i,a}$ coincide in the zero-vortex sector Φ_0:

Lemma S5.2. The action of paraparticle creation and annihilation operators $\hat{\psi}^{\pm}_{i,a}(P)$ in the zero-vortex sector does not depend on the path P, i.e. $\hat{\psi}^{\pm}_{i,a}(P_1)\left|\Psi\right\rangle = \hat{\psi}^{\pm}_{i,a}(P_2)\left|\Psi\right\rangle, \forall \left|\Psi\right\rangle \in \Phi_0$.

Proof. Let $|G\rangle \in \Phi_0$ be the ground state of $\hat{H}_1$ with $n_i = 0$ on every site. It can be shown that the action of $\hat{\psi}^{+}_{i,a}$ on $|G\rangle$ reduces to a ribbon operator in Kitaev's quantum double model [46, 53], and is known to be path independent [46] (we also verify this computationally on a small lattice in the accompanying code [45]), i.e. $\hat{\psi}^{+}_{i,a}\left|G\right\rangle = \hat{\psi}'^{+}_{i,a}\left|G\right\rangle$. Then $\hat{\psi}^{+}_{i,a} - \hat{\psi}'^{+}_{i,a}$ annihilates all other states in the zero vortex sector as well, since, due to Fact S5.4, all other states in the zero-vortex sector can be created by applying products of $\hat{\psi}^{+}_{i,a}$ on $|G\rangle$, and due to the CR in Lemma S5.1, $\hat{\psi}^{+}_{i,a} - \hat{\psi}'^{+}_{i,a}$ can be moved all the way to the right to annihilate $\prod_{i,a}\hat{\psi}^{+}_{i,a}\left|G\right\rangle$. Therefore, $\hat{\psi}^{+}_{i,a} = \hat{\psi}'^{+}_{i,a}$ in the zero vortex sector, and by taking Hermitian conjugate, $\hat{\psi}^{-}_{i,a} = \hat{\psi}'^{-}_{i,a}$ as well. □

From the definition of the two paths P_1 and P_2 shown in Fig. S7, we see that every edge of the lattice either lies on P_1 or P_2 (or both). From Fact S5.3, if an edge $\langle ij\rangle \in P_1$, we have $\hat{h}_{ij} = \sum_a J_{ij}\hat{\psi}^{+}_{j,a}\hat{\psi}^{-}_{i,a} + \text{h.c.}$ Otherwise $\langle ij\rangle \in P_2$, and we have $\hat{h}_{ij} = \sum_a J_{ij}\hat{\psi}'^{+}_{j,a}\hat{\psi}'^{-}_{i,a} + \text{h.c.}$ But Lemma S5.2 claims that the actions of $\{\hat{\psi}^{+}_{i,a}\}$ and $\{\hat{\psi}'^{-}_{i,a}\}$ on the zero-vortex sector are exactly the same, therefore, we conclude that in the zero-vortex sector, every 3-body term $\hat{h}_{ij}$ is mapped to $\sum_a J_{ij}\hat{\psi}^{+}_{j,a}\hat{\psi}^{-}_{i,a} + \text{h.c.}$, completing the proof of Thm. 1.

D. Creation and measurement of the paraparticles

When we describe the paraparticle exchange process in Methods, we claim that the paraparticles in the 2D solvable spin model can be locally created and measured at the upper left and lower right corners of the 2D lattice with OBC, as shown in Fig. 2. We prove this claim in the following. We first prove that paraparticles can be locally created at the two corners (sites i and j in Fig. 2) by applying local operators $y^{+}_{i,a}, y^{+}_{j,b}$ to the ground state $|G\rangle$. More precisely, we prove the identity

$$\hat{y}^{+}_{i,a}\hat{y}^{+}_{j,b}\left|G\right\rangle = \hat{\psi}^{+}_{i,a}\hat{\psi}^{+}_{j,b}\left|G\right\rangle \equiv |G; ia, jb\rangle. \tag{S40}$$

From Eq. (34), we see that $\hat{\psi}^{+}_{i,a} = \hat{y}^{+}_{i,a}$, and $\hat{\psi}^{+}_{j,b} = \sum_c \hat{W}_{bc}\hat{y}^{+}_{j,c}$, where $\hat{W}_{bc}$ is the MPO Jordan-Wigner string connecting sites i and j, so we only need to prove that $\hat{W}_{bc}\left|G\right\rangle = \delta_{bc}\left|G\right\rangle$ (note that $[\hat{W}_{bc}, \hat{y}^{+}_{j,c}] = 0$). From Fact S5.4, we know that $\hat{W}_{bc}$ commutes with all the loop terms $\hat{A}_\nu, \hat{B}_p$ in $\hat{H}_1$, therefore $\hat{W}_{bc}\left|G\right\rangle$ is still a ground state of $\hat{H}_1$. Furthermore, it is straightforward to check that $[\hat{n}, \hat{W}_{bc}] = 0$, leading to $\hat{n}\hat{W}_{bc}\left|G\right\rangle = 0$, i.e., $\hat{W}_{bc}\left|G\right\rangle$ has no paraparticles. It follows that $\hat{W}_{bc}\left|G\right\rangle$ is also a ground state of the system. Since the ground state is unique in OBC shown in Fig. 2 (Fact. S5.2), we have $\hat{W}_{bc}\left|G\right\rangle = W_{bc}\left|G\right\rangle$, where W_{bc} are some constant numbers. It can be proved that $W_{bc} = \delta_{bc}$ [54]. Therefore Eq. (S40) holds.

Since we assumed in the Methods that the ground state $|G\rangle$ has no paraparticles, i.e., $\hat{n}_l\left|G\right\rangle = 0$ at any black site l, the qudit at l is disentangled from the rest of the system and is in the state $|0\rangle$. At any black site l, we can find local unitary operators $\hat{U}_a$ that "implement" the action of $\hat{y}^{+}_a$, i.e., $\hat{U}_a\left|0\right\rangle = \hat{y}^{+}_a\left|0\right\rangle$, leading to $\hat{U}_{l,a}\left|G\right\rangle = \hat{y}^{+}_{l,a}\left|G\right\rangle$. Therefore, we have $|G; ia, jb\rangle = \hat{y}^{+}_{i,a}\hat{y}^{+}_{j,b}\left|G\right\rangle = \hat{U}_{i,a}\hat{U}_{j,b}\left|G\right\rangle$, i.e., the two paraparticle state $|G; ia, jb\rangle$ can be created by applying local unitary operators at the two corners.

We now prove that the paraparticle indices a', b' can be locally measured at the two corner sites i and j in the final state $|G; ib', ja'\rangle = \hat{\psi}^{+}_{i,b'}\hat{\psi}^{+}_{j,a'}\left|G\right\rangle = \hat{y}^{+}_{i,b'}\hat{y}^{+}_{j,a'}\left|G\right\rangle$. At any black site l, we define the local operator

$$\hat{c}_l = \sum_{c=1}^{4} c\ \hat{y}^{+}_{l,c}\hat{y}^{-}_{l,c}. \tag{S41}$$

The result of measuring $\hat{c}_i$ at site i is computed as

$$\begin{aligned}\hat{c}_i\left|G; ib', ja'\right\rangle &= \sum_c c\ \hat{y}^{+}_{i,c}\hat{y}^{-}_{i,c}\hat{y}^{+}_{i,b'}\hat{y}^{+}_{j,a'}\left|G\right\rangle \\ &= b'\ \hat{y}^{+}_{i,b'}\hat{y}^{+}_{j,a'}\left|G\right\rangle \\ &= b'\left|G; ib', ja'\right\rangle,\end{aligned} \tag{S42}$$

where we used the CR in Eq. (S21) and the commutativity between $\hat{y}^{-}_{i,c}$ and $\hat{y}^{+}_{j,a'}$, and that $\hat{y}^{-}_{i,c}\left|G\right\rangle = 0$, $\forall c$. Therefore, measuring $\hat{c}_i$ at site i gives a definite result b'. Similarly, measuring $\hat{c}_j$ at site j gives a'. This completes the proof.

S6. SUPERSELECTION RULES AND THE OBSERVABILITY OF PARASTATISTICS

As we mentioned at the end of the main text, it is straightforward to incorporate relativity into our second quantized theory to get a relativistic quantum field theory of elementary paraparticles. Nevertheless, in such a theory there are superselection rules that fundamentally constrain the observability of the exclusion and exchange statistics of paraparticles. In this section we explain this

issue in detail, and then discuss how our proposed realization of emergent paraparticles in condensed matter systems breaks these superselection rules, which motivates routes to construct theories of elementary paraparticles that are observably distinct from fermions and bosons, in a way evading the no-go theorems [55].

Superselection rules arise because the full state space of our second quantized theory is a direct sum of exponentially many subspaces, such that any physical observable has zero matrix element between states of different subspaces. Each subspace is called a superselection sector. An immediate consequence of the superselection rules is that they forbid quantum transitions (by any local unitary evolution) and thermalization between different superselection sectors. If the system is initialized in one sector, it will stay in that sector forever. This prevents the distinct thermodynamics of free paraparticles (and hence their exclusion statistics) to be physically observed, since the correct thermodynamic description of the system in equilibrium is through the partition function $Z_\pi = \mathrm{Tr}_\pi[e^{-\beta\hat{H}}]$, where Tr_π means summing over all states in a specific sector π, and the result is generally different from that in Fig. 1 obtained by averaging over the whole space (all sectors). In our second quantized theory of paraparticles, it can be shown that the thermal expectation values of all physical observables in a specific sector are the same as some system of ordinary fermions and bosons, meaning that paraparticles in our second quantized theory cannot be distinguished from ordinary particles by local measurements. This is reminiscent of the famous Doplicher-Haag-Roberts (DHR) no-go theorem [55], which states, roughly, that any given superselection sector of a paraparticle system is equivalent to a given fixed particle number sector of a system of fermions and bosons. This problem does not arise for emergent paraparticles in our quantum spin models defined in Eqs. (17) and (32), which have no such superselection rules, since any two states of the full Hilbert space can be connected by some local spin operators $\{\hat{x}^{\pm}_{i,a}, \hat{y}^{\pm}_{i,a}\}$. The non-trivial exclusion and exchange statistics of paraparticles can therefore be physically observed in our solvable spin models. For example, adding an infinitesimal perturbation by such local operators $\{\hat{x}^{\pm}_{i,a}, \hat{y}^{\pm}_{i,a}\}$ will induce thermalization between sectors without perturbing the thermodynamic behavior, allowing the distinct thermodynamics of free paraparticles shown in Fig. 1 to be physically observed. This is similar to how interactions are necessary to thermalize an ideal gas. Similarly, as we demonstrated in Methods and Sec. S5 D, the nontrivial exchange statistics of paraparticles can be physically observed in our 2D spin model, where we exploited the important fact that the local operators $\{\hat{x}^{\pm}_{i,a}, \hat{y}^{\pm}_{i,a}\}$ allow local creation and measurement of paraparticles at the boundary of the system.

The way emergent paraparticles in the quantum spin models in Eqs. (17) and (32) evade the conclusions of the DHR no-go theorem gives us an important hint on the relevance of parastatistics to elementary particles. Despite being a rigorous result, the DHR no-go theorem makes several technical assumptions on the physical systems being considered, one of which is the DHR condition [55, 56], which essentially assumes that all excitations are created by local operators. Yet as we see in Eqs. (18) and (34), the quasiparticles in our spin models are created by non-local string operators, thereby rendering inapplicable the DHR theorem in a similar way as the anyonic excitations in Kitaev's toric code model [46, 57], whose creation operators are attached by Z_2 gauge strings. Although our spin models are non-relativistic, one can potentially introduce other local observables that are compatible with causality and relativistic covariance but which break the superselection rules, like $\{\hat{x}^{\pm}_{i,a}, \hat{y}^{\pm}_{i,a}\}$ in the spin models. A promising direction is to consider paraparticles coupled to gauge fields, as the DHR theorem does not apply to quantum gauge theories [56], such as Kitaev's toric code model (Z_n gauge theory) and Chern-Simons theories [58], where anyons emerge.

[1] H. S. Green, Phys. Rev. **90**, 270 (1953).
[2] C. Nayak, S. H. Simon, A. Stern, M. Freedman, and S. Das Sarma, Rev. Mod. Phys. **80**, 1083 (2008).
[3] P. Fendley, J. Phys. A Math. Theor. **47**, 75001 (2014).
[4] J. Alicea and P. Fendley, Annu. Rev. Condens. Matter Phys. **7**, 119 (2016).
[5] H. Araki, J. Math. Phys. **2**, 267 (1961).
[6] O. W. Greenberg and A. M. L. Messiah, Phys. Rev. **138**, B1155 (1965).
[7] P. Landshoff and H. P. Stapp, Ann. Phys. **45**, 72 (1967).
[8] K. Drühl, R. Haag, and J. E. Roberts, Commun. Math. Phys. **18**, 204 (1970).
[9] R. H. Stolt and J. R. Taylor, Nucl. Phys. B **19**, 1 (1970).
[10] N. I. Stoilova and J. Van der Jeugt, Phys. Lett. A **384**, 126421 (2020).
[11] B. Aneva and T. Popov, J. Phys. A. Math. Gen. **38**, 6473 (2005).
[12] K. Kanakoglou and C. Daskaloyannis, J. Math. Phys. **48**, 113516 (2007).
[13] C. H. Alderete and B. M. Rodriguez-Lara, Phys. Rev. A **95**, 013820 (2017).
[14] C. H. Alderete, A. M. Green, N. H. Nguyen, Y. Zhu, B. M. Rodriguez-Lara, and N. M. Linke, arXiv Prepr. arXiv2108.05471 (2021).
[15] F. Toppan, J. Phys. A Math. Theor. **54**, 115203 (2021).
[16] F. Toppan, J. Phys. A Math. Theor. **54**, 355202 (2021).
[17] L. C. Biedenharn, J. Phys. A. Math. Gen. **22**, L873 (1989).
[18] G. Gentile j., Nuovo Cim. **17**, 493 (1940).
[19] F. D. M. Haldane, Phys. Rev. Lett. **67**, 937 (1991).
[20] R. B. Laughlin, Phys. Rev. Lett. **50**, 1395 (1983).
[21] K. Schoutens, Phys. Rev. Lett. **79**, 2608 (1997).
[22] P. Bouwknegt and K. Schoutens, Nucl. Phys. B **547**, 501 (1999).

[23] N. M. Sánchez and B. Dakić, Quantum **8**, 1473 (2024).

[24] First, notice that $\hat{\psi}^{+}_{i,a} = (\hat{\psi}^{-}_{i,a})^{\dagger}$ is fully consistent with the fundamental CRs in Eq. (6), as taking the Hermitian conjugate on both sides leaves Eq. (6) invariant (maps the first line to itself and swaps the second and the third lines). Then it can be checked straightforwardly that the explicit matrix representation of $\hat{\psi}^{\pm}_{i,a}$ defined in Sec. S2 B indeed satisfies $\hat{\psi}^{+}_{i,a} = (\hat{\psi}^{-}_{i,a})^{\dagger}$. By contrast, if R is not unitary, the relation $\hat{\psi}^{+}_{i,a} = (\hat{\psi}^{-}_{i,a})^{\dagger}$ is not consistent with Eq. (6) as taking the Hermitian conjugate on both sides leads to extra relations that result in an algebraic inconsistency, and the representation of $\hat{\psi}^{\pm}_{i,a}$ constructed in Sec. S2 B does not satisfy $\hat{\psi}^{+}_{i,a} = (\hat{\psi}^{-}_{i,a})^{\dagger}$. However, as we show in Sec. S2 D 5, even for a non-unitary R, we can still define a Hermitian inner product on the Fock space such that $\hat{e}^{\dagger}_{ij} = \hat{e}_{ji}$ is satisfied, and consequently all physical observables are Hermitian with respect to this inner product.

[25] A. Polishchuk and L. Positselski, *Quadratic algebras*, Vol. 37 (American Mathematical Society, 2005).

[26] P. Etingof, T. Schedler, and A. Soloviev, Duke Math. J. **100**, 169 (1999).

[27] A. Giaquinto and J. Zhang, J. Algebra **176**, 861 (1995).

[28] S. Majid, Int. J. Mod. Phys. A **05**, 1 (1990).

[29] P. I. Etingof, O. Golberg, S. Hensel, T. Liu, A. Schwendner, D. Vaintrob, and E. Yudovina, *Introduction to representation theory*, Vol. 59 (American Mathematical Society, 2011).

[30] N. Bourbaki, in *Lie groups and Lie algebras: chapters 7-9* (Springer, Berlin Heidelberg, 2005) Chap. 9, pp. 281–377.

[31] J. E. Humphreys, *Introduction to Lie algebras and representation theory* (Springer-Verlag (New York), 1972).

[32] Note that it is exactly for this reason that the Doplicher-Haag-Roberts (DHR) no-go theorem [55] does not apply to the first quantization formulation, since the former takes locality as a fundamental assumption, while the latter does not have locality built-in.

[33] J. B. Hartle and J. R. Taylor, Phys. Rev. **178**, 2043 (1969).

[34] R. H. Stolt and J. R. Taylor, Phys. Rev. D **1**, 2226 (1970).

[35] G. Lechner, U. Pennig, and S. Wood, Adv. Math. **355**, 106769 (2019).

[36] A. Chapman and S. T. Flammia, Quantum **4**, 278 (2020).

[37] The model is still well-defined for $m = 2$, where $\hat{H}$ is Hermitian. But that case is trivial: when $m = 2$, $\hat{H}$ is equal to the sum of two decoupled chains of XY models.

[38] C. M. Bender, Rep. Prog. Phys. **70**, 947 (2007).

[39] R. El-Ganainy, K. G. Makris, M. Khajavikhan, Z. H. Musslimani, S. Rotter, and D. N. Christodoulides, Nat. Phys. **14**, 11 (2018).

[40] D. E. Radford, J. Algebr. **157**, 285 (1993).

[41] S. Majid, *Foundations of Quantum Group Theory* (Cambridge University Press, Cambridge, 1995).

[42] P. Etingof and S. Gelaki, Math. Res. Lett. **5**, 551 (1998).

[43] P. Etingof and S. Gelaki, Int. Math. Res. Not. **2000**, 223 (2000).

[44] The minimal $\mathbb{C}^*$ triangular Hopf algebra $\mathcal{H}_{64}$ is constructed using the method introduced in Ref. [42], and it plays a key role in the construction of the tensors u_L, v_L, u_R, v_R. Our construction of u_L, v_L, u_R, v_R from a minimal triangular Hopf algebra is partially motivated by the relation between MPO and Hopf algebra introduced in Ref. [59]. We here mention that it may also be interesting to realize emergent paraparticles in higher dimensional fermionic systems, where triangular Hopf superalgebras [60, 61] may be useful tools in constructing exactly solvable models (this is partially motivated by the connection between Lie superalgebras [62–64] and Green's parastatistics described in some recent works [15, 16]).

[45] We provide accompanying Mathematica codes for computational verification of several important mathemtical facts in the main text and SI at `https://github.com/lagrenge94/Mathematica-codes-for-parastatistics`. There are two notebook files in total, their goals are: (1) In RMatricesAnd1DSpinModel.nb we construct all the R-matrices that appear in this paper, verify that they satisfy the YBE (5), and compute the exclusion statistics and single mode partition function $z_R(x)$ for each R-matrix; then we construct the local operators of the 1D solvable spin model for each R-matrix, and verify that they satisfy the defining relations in Eq. (S21); (2) In Verify2DSpinModel.nb, we first construct the tensors u_L, u_R, v_L, v_R in the 2D spin model, and verify they satisfy all the CRs in Fig. S5; then we verify Fact. S5.2 that the $\hat{H}_1$ reduces to Kitaev's quantum double model when all black sites are in the state $|0\rangle$, and has a unique ground state on a 3×3 lattice; then we verify that the action of the JW string on the ground state is path independent, as claimed in Fact. S5.2; finally we verify that the JW string connecting upper left and lower right corner acts on the ground state as a delta function $\hat{W}_{bc} |G\rangle = \delta_{bc} |G\rangle$, a property we used with only a partial proof in Sec. S5 D.

[46] A. Kitaev, Ann. Phys. **303**, 2 (2003).

[47] O. Buerschaper and M. Aguado, Phys. Rev. B **80**, 155136 (2009).

[48] S. Beigi, P. W. Shor, and D. Whalen, Commun. Math. Phys. **306**, 663 (2011).

[49] O. Buerschaper, J. M. Mombelli, M. Christandl, and M. Aguado, J. Math. Phys. **54**, 12201 (2013).

[50] C. Meusburger, Commun. Math. Phys. **353**, 413 (2017).

[51] Z. Jia, S. Tan, D. Kaszlikowski, and L. Chang, Commun. Math. Phys. **402**, 3045 (2023).

[52] A. Cowtan and S. Majid, J. Math. Phys. **64**, 102203 (2023).

[53] B. Yan, P. Chen, and S. X. Cui, J. Phys. A Math. Theor. **55**, 185201 (2022).

[54] This is very natural from a physical viewpoint, because $W_{bc} = \delta_{bc}$ means that when there is only one paraparticle excitation, the index b of the paraparticle does not change when we transport it from the upper left to the lower right corner using $\hat{E}_{ij}$: $\hat{y}^{+}_{i,b} |G\rangle = \hat{\psi}^{+}_{i,b} |G\rangle \xrightarrow{\hat{E}_{ij}} \hat{\psi}^{+}_{j,b} |G\rangle = \hat{y}^{+}_{i,b} |G\rangle$. The proof involves technical results about the quantum double ground state, which we do not prove in this paper, but we verify it in a small system in the accompanying code [45].

[55] S. Doplicher, R. Haag, and J. E. Roberts, Commun. Math. Phys. **23**, 199 (1971); Commun. Math. Phys. **35**, 49 (1974).

[56] R. Haag, *Local quantum physics: Fields, particles, algebras* (Springer-Verlag, Berlin, Heidelberg, 1996).

[57] P. Naaijkens, *Quantum spin systems on infinite lattices*, Lecture Notes in Physics (Springer Cham, 2017).

[58] E. Witten, Commun. Math. Phys. **121**, 351 (1989).

[59] A. Molnar, A. Ruiz-de Alarcón, J. Garre-Rubio,

N. Schuch, J. I. Cirac, and D. Pérez-García, arXiv Prepr. arXiv2204.05940 (2022).
[60] N. Andruskiewitsch, P. Etingof, and S. Gelaki, Michigan Math. J. **49**, 277 (2001).
[61] P. Etingof and S. Gelaki, Math. Res. Lett. **8**, 249 (2001).
[62] V. G. Kac, Adv. Math. (N. Y). **26**, 8 (1977).
[63] V. Rittenberg and D. Wyler, Nucl. Phys. B **139**, 189 (1978).
[64] V. Rittenberg and D. Wyler, J. Math. Phys. **19**, 2193 (1978).
